# Prevalence and risk factors for postoperative delirium after hip fracture in the elderly: A systematic review and meta-analysis

**DOI:** 10.1097/MD.0000000000047296

**Published:** 2026-01-23

**Authors:** Yue Hu, Wen Tang, Xiuzhen Jiang

**Affiliations:** a Department of Medicine, Hetao College, Bayannur, Inner Mongolia Autonomous Region, China; b Department of Orthopedics Center, The First Affiliated Hospital, Hengyang Medical School, University of South China, Hengyang, Hunan, China; c Health Care Center, Inner Mongolia Autonomous Region People’s Hospital, Hohhot, Inner Mongolia Autonomous Region, China.

**Keywords:** hip fracture, meta-analysis, postoperative delirium, risk factors

## Abstract

**Background:**

The aim of this study was to systematically assess the prevalence and risk factors for hip fracture postoperative delirium (POD) in elderly patients.

**Methods:**

PubMed, Embase, Cochrane Library, and other databases were searched to identify observational studies on risk factors for POD in patients with hip fracture, from database inception to April 1, 2025. Two independent reviewers conducted literature screening and data extraction, with disagreements resolved by discussion or consultation with a 3rd reviewer. The quality of the included studies was assessed using the Newcastle–Ottawa Scale. Meta-analysis was performed using Stata 15.0 software. Heterogeneity among studies was evaluated using the *I*² statistic and Cochran *Q* test. A random-effects model was applied when substantial heterogeneity was present (*I*² > 50% or *P* < .10), otherwise a fixed-effects model was used. In addition, subgroup analyses were conducted when appropriate to explore potential sources of heterogeneity.

**Results:**

A total of 13 cohort studies (N = 18,850) were included, all the articles included in this study were of high quality. Meta-analysis results suggest that prevalence of POD after hip fracture was (effect size = 23%, 95% confidence intervals [CI] [19–28%]), age > 80 (odds ratios [OR] = 1.14, 95% CI [1.08–1.21]), ASA classification > 4 (OR = 4.19, 95% CI [1.85–9.52]); dementia (OR = 3.42, 95% CI [2.32–5.05]); diabetes (OR = 1.66, 95% CI [1.05–2.62]); general anesthesia (OR = 1.53, 95% CI [1.09–2.15]); hypertension (OR = 1.57, 95% CI [1.17–2.12]); male (OR = 1.23, 95% CI [1.12–1.35]); prior history of delirium (OR = 6.44, 95% CI [2.95–14.06]) were a risk factor for POD in elderly patients with hip fracture.

**Conclusion:**

In this study, we assessed the relationship between multiple factors and pod in elderly hip arthroplasty by meta-analysis, which showed that age >80 years, ASA score >4, dementia, diabetes mellitus, general anesthesia, hypertension, being male, and a history of delirium were all risk factors for POD. These findings highlight the importance of perioperative management in high-risk elderly patients.

## 1. Introduction

The elderly population is one of the most rapidly growing demographic groups in the world, and as they age, they face an increasing number of health problems.^[1]^ Among them, hip fracture, a common traumatic fracture in the elderly, has become an important public health problem among the global elderly population. Hip fractures not only have a serious impact on the quality of life of patients but also pose a significant socioeconomic burden.^[2]^ Studies have shown that the incidence of hip fracture in the elderly increases with age, and the incidence of hip fracture in female patients is significantly higher than that in males. After hip fracture, patients not only face a long recovery process, but also may be aggravated by postoperative complications, which may affect their prognosis.^[3,4]^

Postoperative delirium (POD), a common acute cognitive disorder, occurs widely in the elderly population, especially in patients who have undergone major surgery.^[5]^ Studies^[6,7]^ have shown that the incidence of delirium after hip fracture surgery is high, approximately 20% to 50%, and that the risk of POD further increases with age, deterioration of preoperative health, and the presence of a variety of other factors. The clinical manifestations of POD are usually characterized by acute impairment of consciousness, cognitive decline, disorientation, and mood swings, which, in severe cases, can lead to long-term cognitive decline, physical weakness, and a significant reduction in the patient’s quality of life.^[8,9]^

Although the dangers of POD have received widespread attention, studies on its specific risk factors are still incomplete. Existing literature suggests that the development of POD may be closely related to a variety of factors, including the patient’s age, underlying disease, preoperative cognitive function, nutritional status, anesthetic modality, and postoperative complications.^[10,11]^ Although some single studies have pointed out the correlation between these factors and pod, the findings of these studies are often controversial and inconsistent due to differences in study design and limitations in sample size.^[12,13]^ Currently, there is a lack of large-sample meta-analyses quantifying the relative contributions of different risk factors. Therefore, it is important for clinical practice to identify and quantify these risk factors.

Most of the existing studies are single-center studies with limited sample sizes, and the findings are often geographically diverse, making it difficult to provide conclusions that are widely applicable.^[14,15]^ To solve this problem, this study comprehensively analyzed all the relevant literature through systematic evaluation and meta-analysis, to reveal the main risk factors for delirium in the elderly after hip fracture surgery, and to provide a more accurate risk assessment basis for the clinic.^[16]^ Although several meta-analyses^[17–19]^ have explored risk factors for POD following fracture surgery, the published studies are relatively dated. Recently, numerous high-quality studies have emerged, some employing univariate analyses where confounding factors may have influenced outcomes. Consequently, this study incorporated the most recent articles, covering both Chinese and English databases. It assessed the prevalence of POD, conducted subgroup analyses, and utilized multivariate regression for risk factor analysis, yielding more reliable results.

The purpose of this study is to sort out and summarize the prevalence and risk factors for delirium in the elderly after hip fracture surgery, explore their potential clinical risk factors, and assess the contribution of different risk factors to the occurrence of POD through systematic evaluation and meta-analysis. By comprehensively analyzing these factors, we expect to provide clinicians with a scientific and reasonable risk prediction tool, which will in turn optimize postoperative management, reduce the occurrence of postoperative complications, and improve patients’ prognosis and quality of life.

## 2. Methods

This systematic evaluation and meta-analysis will strictly follow the Preferred Reporting Items for Systematic Reviews and Meta-Analyses (PRISMA) guidelines.^[20]^ And it is registered in Prospero with registration number CRD420251010651.

### 2.1. Inclusion and exclusion criteria

Inclusion criteria were: the type of study was original, including observational studies (cohort, case–control, and cross-sectional); the study was conducted on patients aged 65 years and older who underwent surgical treatment for hip fracture; the study focused on the association between the development of pod and multiple factors (age, gender, underlying disease, anesthesia modality, and preoperative cognitive status); and the study explicitly use standardized diagnostic tools for delirium (CAM-ICU, DSM-5).

Exclusion criteria were: non-original studies such as review articles, commentary articles, letters, case reports, and case series reports; studies of patients without a definitive diagnosis of hip fracture, or where the surgical procedure was unclear; studies for which relevant data could not be provided; studies involving non-elderly populations (<65 years of age) or other types of fractures (non-fractures of the hip, femur fractures); and studies where the data were incomplete, where it was not possible to be analyzed, or studies with serious flaws in statistical methods and unclear data presentation.

### 2.2. Literature retrieval

Observational studies of risk factors for POD in hip fracture patients were screened by searching databases such as PubMed, Embase, and Cochrane Library from inception to April 1, 2025. The search terms were Aged, Hip Fractures, Pod, Risk Factor, and the specific search strategy is described in Table S1, Supplemental Digital Content, https://links.lww.com/MD/R240.

### 2.3. Data extractions

Two authors independently screened the literature for inclusion by importing the literature into endnote according to the literature inclusion and exclusion criteria, the final included studies were used for data extraction using Excel software and if there was a dispute about the literature screening then it would be discussed, or a 3rd person would be sought to adjudicate. The extracted data contained basic characteristics of the study (1st author, year of publication, study design, country), basic characteristics of the population (sample size, gender, number of deliriums, mean age) and regression model.

### 2.4. Risk of bias

The Newcastle–Ottawa Scale (NOS)^[21]^ was used in this study to evaluate the quality of the included observational studies. The scale is categorized into 3 main dimensions based on the type of study (cohort study or case–control study): selection of study subjects (0–4 points), comparability of comparison groups (0–2 points), and outcome measures (0–3 points) out of a possible 9 points. A score of ≥7 is considered high quality, 5 to 6 is considered moderate quality and ≤4 is considered low quality. The 2 evaluators will score independently, and any disagreements in scoring will be resolved through negotiation or referred to a third party for validation.

### 2.5. Statistical analysis

This study employed Stata 15.0 software (College Station) for meta-analysis. All pooled effect estimates derived from the included studies were obtained via multivariate regression analysis (multivariate regression) to calculate odds ratios (OR) and their 95% confidence intervals (CI). This methodology enhances analytical precision by controlling for potential confounding factors. OR were pooled using inverse variance weighting. Heterogeneity was assessed via Cochran *Q* test and the *I*² statistic. A fixed-effects model was employed when *I*² < 50%, indicating low heterogeneity; a random-effects model was used when *I*² ≥ 50%, signaling substantial heterogeneity warranting further investigation into its sources. Subgroup analyses were conducted by country to explore potential differences in effects across groups. Sensitivity analyses were performed to assess result robustness by sequentially excluding individual studies to observe outcome stability, thereby evaluating each study’s impact on overall results. Additionally, meta-regression analyses were conducted to investigate how study characteristics (sample size, design) influenced observed effect sizes. Meta-regression analysis aids in identifying factors potentially contributing to inter-study heterogeneity and provides a more comprehensive understanding of the relationship between interventions and outcomes. The meta-regression model employed a random-effects model for analysis, with results used to explore significant factors influencing effect sizes. Finally, funnel plots and Egger test were used to assess publication bias, detecting any asymmetry that might indicate bias in the included studies.

## 3. Results

### 3.1. Literature search results

**Figure 1.**
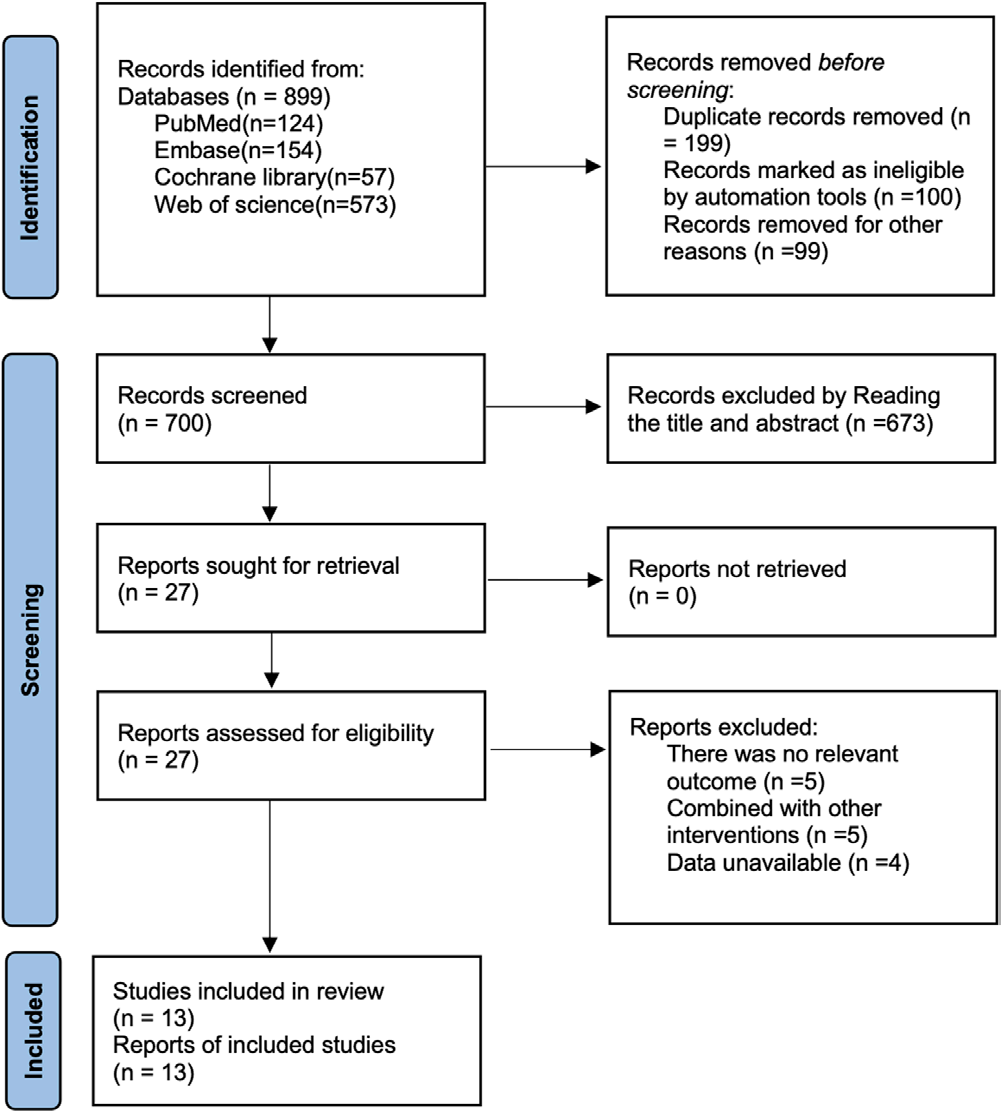
Flowchart of literature retrieval.

A total of 899 articles were retrieved by searching PubMed (n = 124), Embase (n = 154), Cochrane Library (n = 57), and Web of Science (n = 573), and a total of 199 duplicates were removed by removing 199 duplicates, 673 documents were removed by reading the titles and abstracts, and 12 were removed by reading the full text, and finally 15 studies^[12,15,22–32]^ were included. The literature search flowchart is shown in Figure 1.

### 3.2. Basic characteristics of the included literature

A total of 13 cohort studies involving 18,850 patients were included, including 2 studies from the USA and 9 from China, with 11,450 patients experiencing pod. The mean age was 65 years at the minimum and 84 years at the maximum, and the multifactorial regression model used in all of them, and the specific basic characteristics are shown in Table 1.

**Table 1:** Basic characteristics.

Study	Year	Study design	Country	Sample size	Gender (M/F)	Number of delirium	Mean age	Regression model
Ahmed	2022	Cohort study	USA	13,174	9188/4256	3924	84	Multivariable model
Aldwikat	2020	Cohort study	Australia	260	83/177	63	82.1	Multivariable model
An	2023	Cohort study	China	648	256/392	115	70.32	Multivariable model
Chu	2021	Cohort study	China	462	137/315	74	67.5	Multivariable model
Feng	2024	Cohort study	China	148	41/107	16	81	Multivariable model
Guo	2016	Cohort study	China	572	206/366	120	78.1	Multivariable model
Harris	2019	Cohort study	USA	1261	332/929	526	80.2	Multivariable model
He	2022	Cohort study	China	780	379/401	182	74.5	Multivariable model
Juliebo	2009	Cohort study	Norway	364	88/276	50	84	Multivariable model
Mi	2024	Cohort study	China	369	265/104	67	78.4	Multivariable model
Qi	2024	Cohort study	China	234	180/54	114	81.20	Multivariable model
Wang	2018	Cohort study	China	306	104/202	59	77.8	Multivariable model
Wang	2021	Cohort study	China	272	78/194	52	77.9	Multivariable model

### 3.3. Quality evaluation

The NOS score was used in this study and 5 studies^[22,24,26,28,29]^ scored 9, 5 studies^[12,15,23,25,30]^ scored 8, and 3 studies^[27,31,32]^ scored 7, all of which were high-quality studies. The specific quality evaluation results are shown in Table 2.

**Table 2:** NOS scores.

Cohort study									
Study	Representativeness of the exposed group	Selection of non-exposed groups	Determination of exposure factors	Identification of outcome indicators not yet to be observed at study entry	Comparability of exposed and unexposed groups considered in design and statistical analysis	design and statistical analysis	Adequacy of the study’s evaluation of the outcome	Adequacy of follow-up in exposed and unexposed groups	Total scores
Ahmed^[22]^	*	*	*	*	**	*	*	*	9
Aldwikat^[23]^	*	*	*	*	*	*	*	*	8
An^[24]^	*	*	*	*	**	*	*	*	9
Chu^[25]^	*	*	*	*	*	*	*	*	8
Feng^[26]^	*	*	*	*	**	*	*	*	9
Guo^[27]^	*	*	*	–	*	*	*	*	7
Harris^[28]^	*	*	*	*	**	*	*	*	9
He^[29]^	*	*	*	*	**	*	*	*	9
Juliebo^[30]^	*	*	*	*	*	*	*	*	8
Mi^[15]^	*	*	*	*	*	*	*	*	8
Qi^[12]^	*	*	*	*	*	*	*	*	8
Wang^[31]^	*	*	*	–	*	*	*	*	7
Wang^[32]^	*	*	*	–	*	*	*	*	7

NOS = Newcastle–Ottawa Scale.

* Means 1 scores.

** Means 2 points.

### 3.4. Results of meta-analysis

#### 3.4.1. Prevalence

In the current study, the prevalence of POD after hip fracture was assessed by including studies, and the test for heterogeneity (*I*^2^ = 100%, *P* = .001) and the results of the meta-analysis (Figure S1, Supplemental Digital Content, https://links.lww.com/MD/R240) suggested that the prevalence of pod after hip fracture was (effect size [ES] = 23%, 95% CI [19–28%]), and due to the large heterogeneity, the national subgroup analysis was performed, and the results of the analysis (Figure S2, Supplemental Digital Content, https://links.lww.com/MD/R240) suggested a prevalence for the USA (ES = 36%, 95% CI [24–47%]) and for China (ES = 22%, 95% CI [18–25%]). We also used meta-regression to explore sources of heterogeneity (including year, country, sample size, and age), and the results (Table S2, Supplemental Digital Content, https://links.lww.com/MD/R240) suggest that year, country, sample size, and age are not sources of heterogeneity.

#### 3.4.2. Age > 80

**Figure 2.**
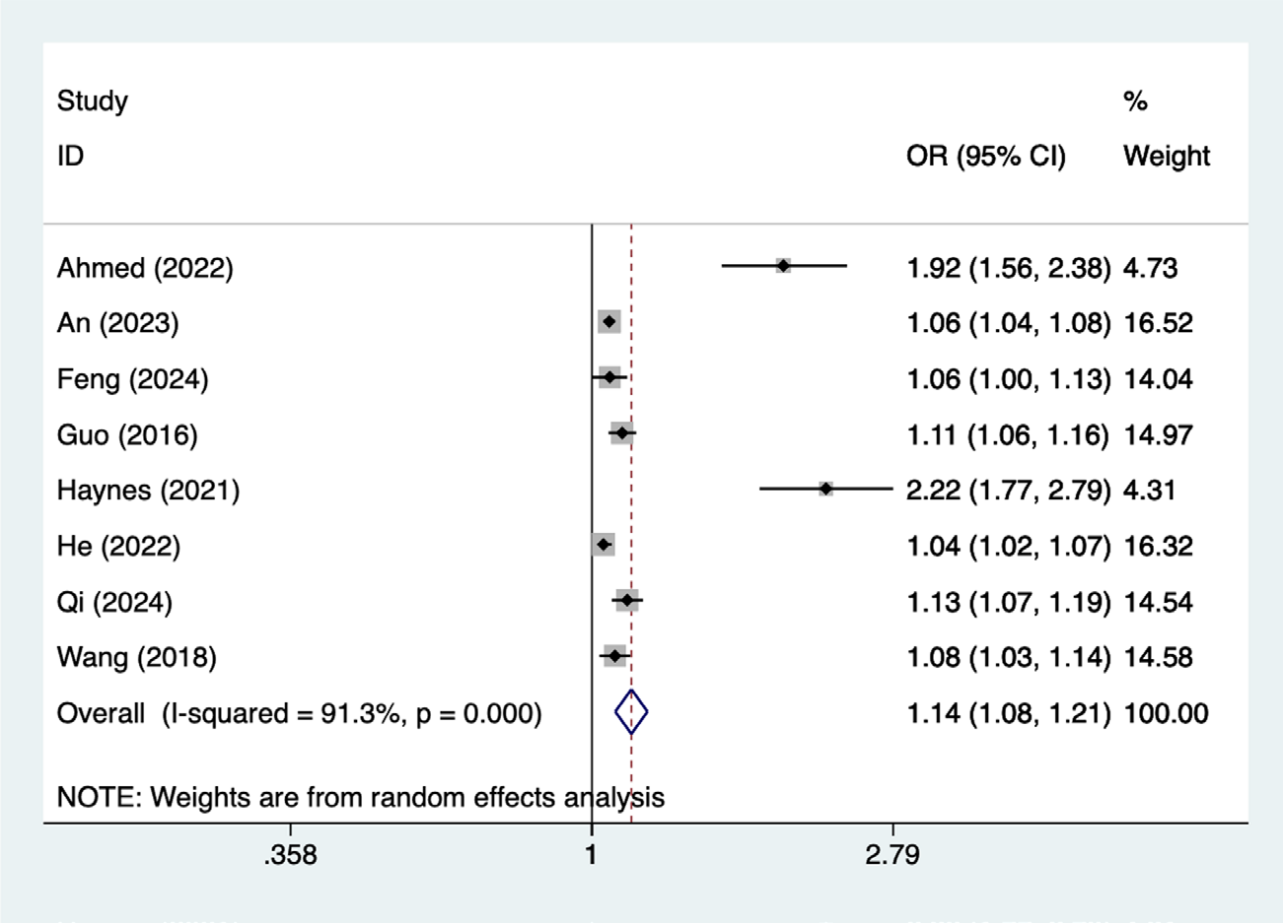
Forest plot of meta-analysis for age > 80.

In the current study, 8 studies mentioned age > 80, the test of heterogeneity (*I*^2^ = 91.5%, *P* = .001), and were analyzed using a random-effects model, and the results of the analysis (Fig. 2) suggested that age > 80 was a risk factor for pod in elderly patients with hip fracture (OR = 1.14, 95% CI [1.08–1.21]), due to the large heterogeneity of the indicators, sensitivity analysis was performed and the analysis results (Figure S3, Supplemental Digital Content, https://links.lww.com/MD/R240) suggested that minimal influence on the results.

#### 3.4.3. ASA classification > 4

**Figure 3.**
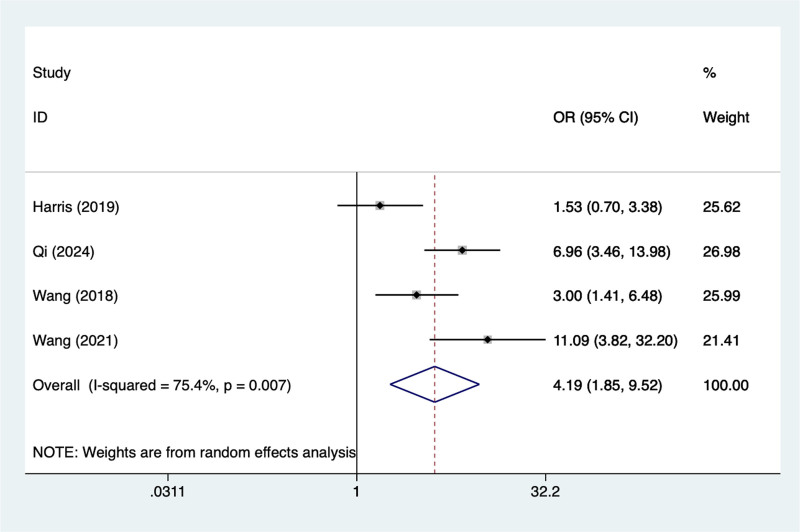
Forest plot of meta-analysis for ASA classification > 4.

In the current study, 4 studies mentioned ASA classification > 4,the test of heterogeneity (*I*^2^ = 88.7%, *P* = .001), and were analyzed using a random-effects model, and the results of the analysis (Fig. 3) suggested that ASA classification > 4 was a risk factor for pod in elderly patients with hip fracture (OR = 4.19, 95% CI [1.85–9.52]), due to the large heterogeneity of the indicators, sensitivity analysis was performed and the analysis results (Figure S4, Supplemental Digital Content, https://links.lww.com/MD/R240) suggest that minimal influence on the results.

#### 3.4.4. Dementia

**Figure 4.**
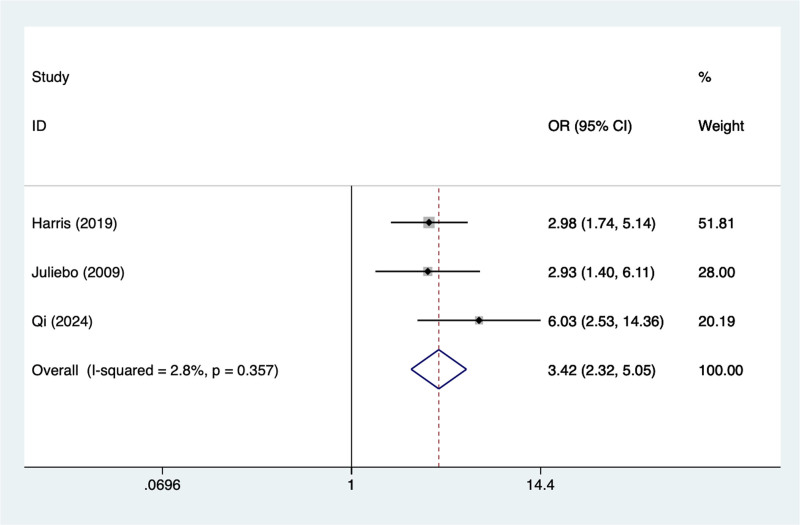
Forest plot of meta-analysis for dementia.

In the current study, 3 studies mentioned dementia, the test of heterogeneity (*I*^2^ = 2.8%, *P* = .357), and were analyzed using a fixed-effects model, and the results of the analysis (Fig. 4) suggested that dementia was a risk factor for pod in elderly patients with hip fracture (OR = 3.42, 95% CI [2.32–5.05]).

#### 3.4.5. Diabetes

**Figure 5.**
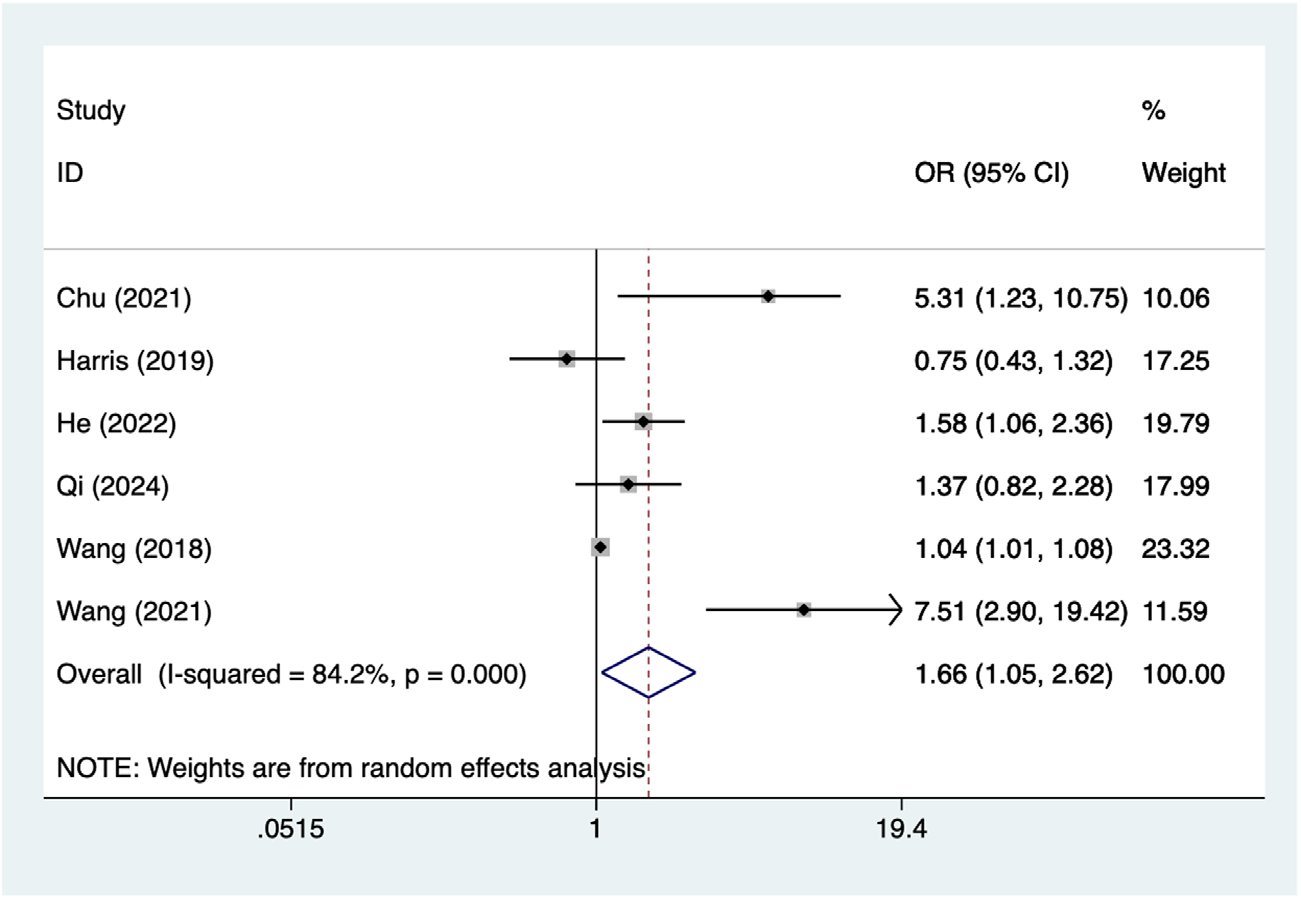
Forest plot of meta-analysis for diabetes.

In the current study, 6 studies mentioned diabetes, the test of heterogeneity (*I*^2^ = 84.2%, *P* = .001), and were analyzed using a random-effects model, and the results of the analysis (Fig. 5) suggested that diabetes was a risk factor for pod in elderly patients with hip fracture (OR = 1.66, 95% CI [1.05–2.62]), due to the large heterogeneity of the indicators, sensitivity analysis was performed and the analysis results (Figure S5, Supplemental Digital Content, https://links.lww.com/MD/R240) suggested that minimal influence on the results.

#### 3.4.6. General anesthesia

**Figure 6.**
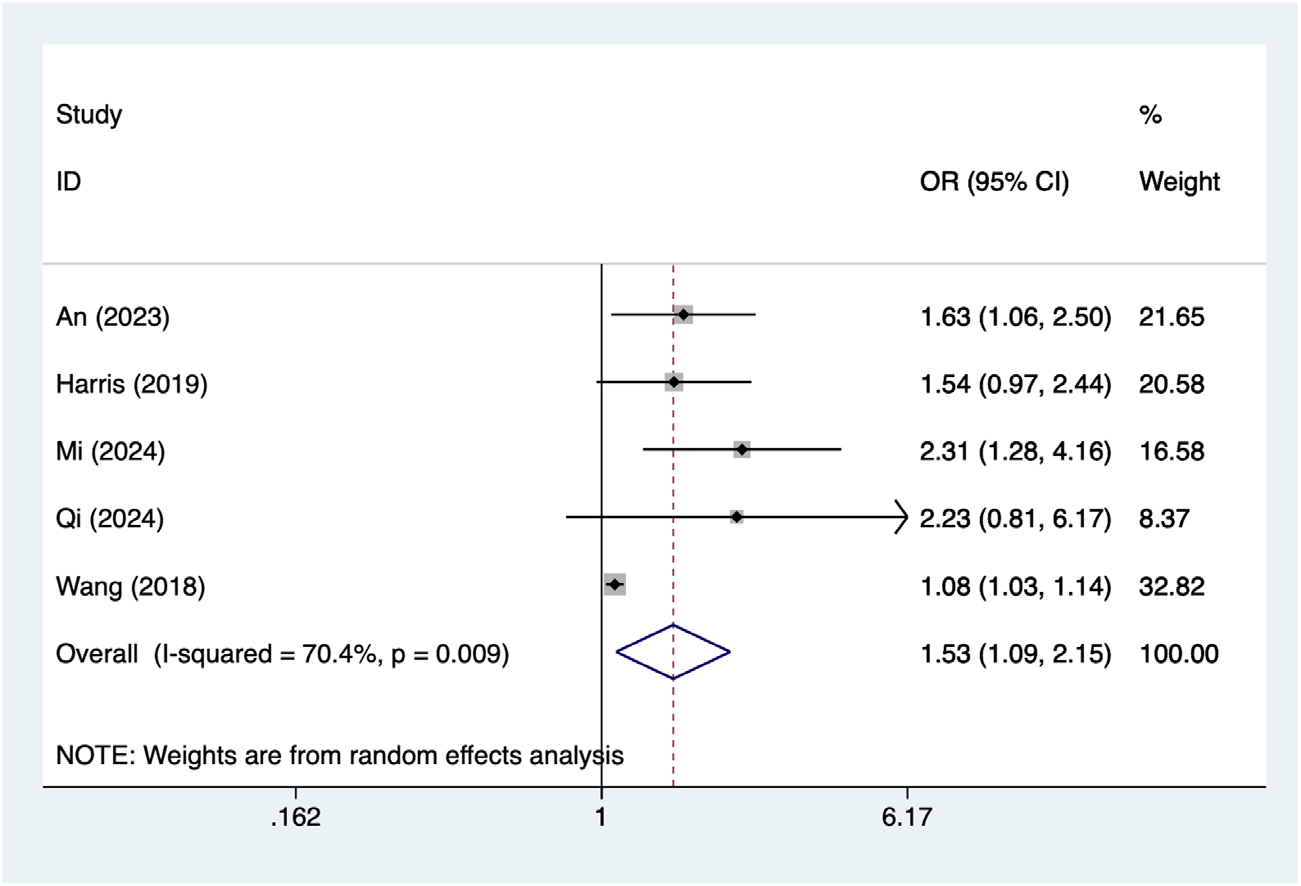
Forest plot of meta-analysis for general anesthesia.

In the current study, 5 studies mentioned general anesthesia, the test of heterogeneity (*I*^2^ = 70.4%, *P* = .009), and were analyzed using a random-effects model, and the results of the analysis (Fig. 6) suggested that general anesthesia was a risk factor for pod in elderly patients with hip fracture (OR = 1.53, 95% CI [1.09–2.15]), due to the large heterogeneity of the indicators, sensitivity analysis was performed and the analysis results (Figure S6, Supplemental Digital Content, https://links.lww.com/MD/R240) suggested that minimal influence on the results.

#### 3.4.7. Hypertension

**Figure 7.**
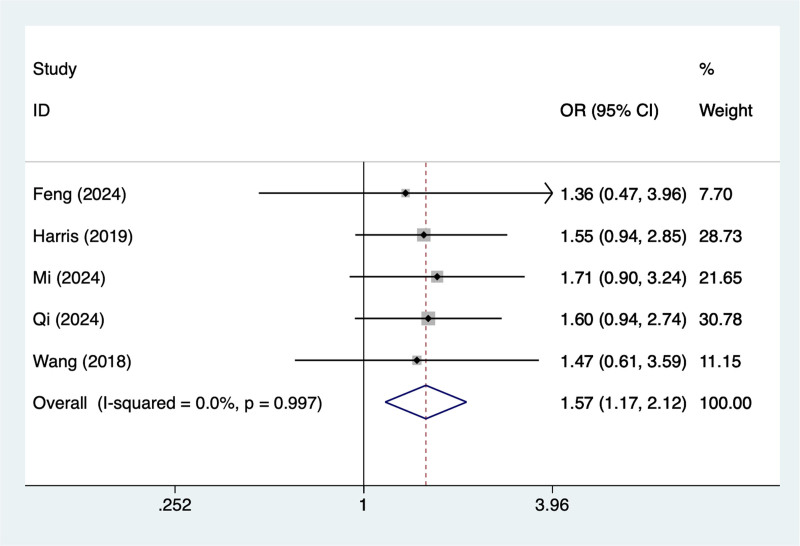
Forest plot of meta-analysis for hypertension.

In the current study, 5 studies mentioned hypertension, the test of heterogeneity (*I*^2^ = 0%, *P* = .997), and were analyzed using a random-effects model, and the results of the analysis (Fig. 7) suggested that hypertension was a risk factor for pod in elderly patients with hip fracture (OR = 1.57, 95% CI [1.17–2.12]).

#### 3.4.8. Male

**Figure 8.**
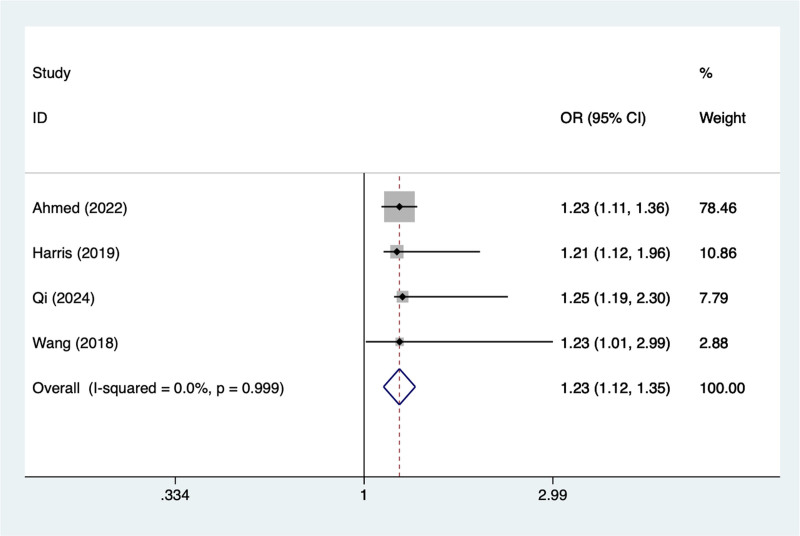
Forest plot of meta-analysis for male.

In the current study, 4 studies mentioned male, the test of heterogeneity (*I*^2^ = 0%, *P* = .999), and were analyzed using a random-effects model, and the results of the analysis (Fig. 8) suggested that male was a risk factor for pod in elderly patients with hip fracture (OR = 1.23, 95% CI [1.12–1.35]).

#### 3.4.9. Prior history of delirium

**Figure 9.**
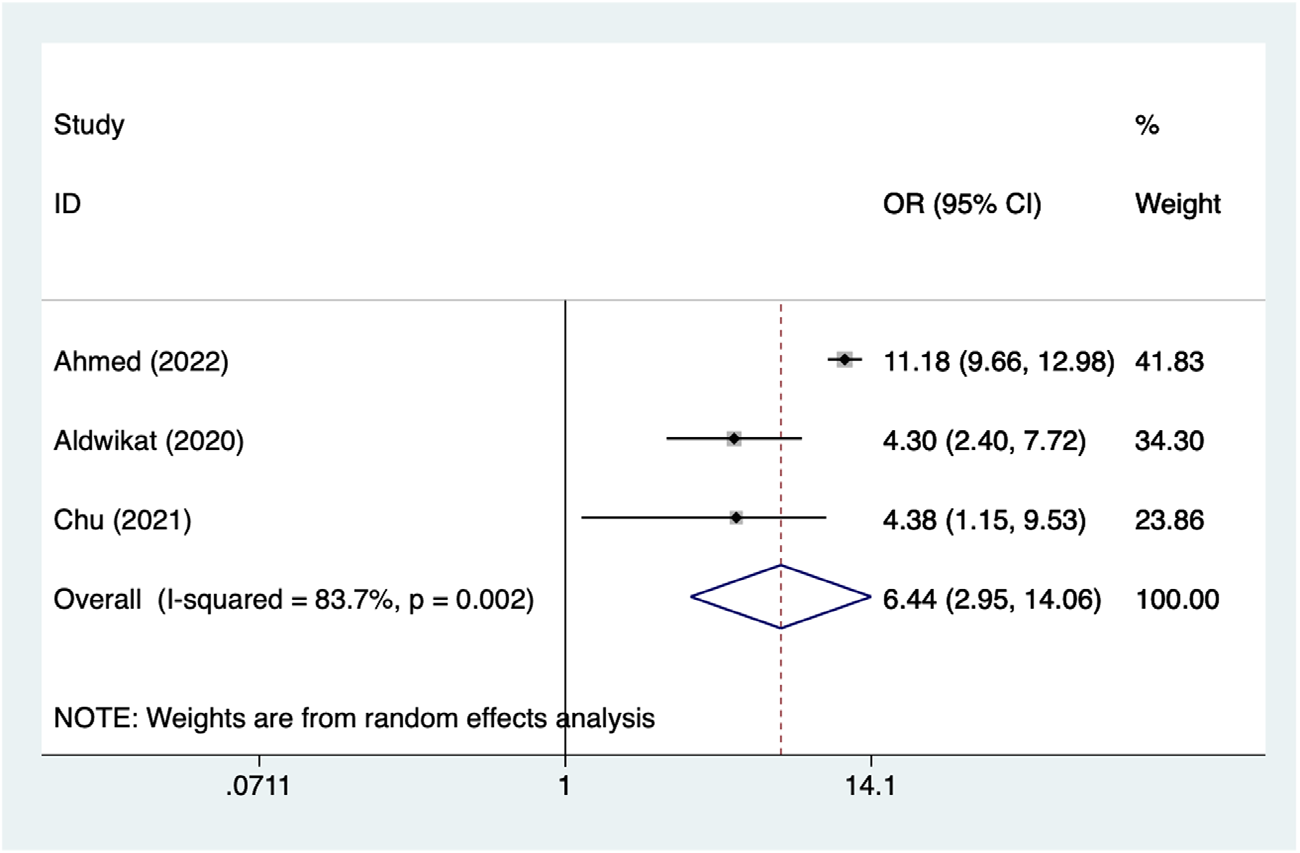
Forest plot of meta-analysis for prior history of delirium.

In the current study, 3 studies mentioned prior history of delirium, the test of heterogeneity (*I*^2^ = 83.7%, *P* = .002), and were analyzed using a random-effects model, and the results of the analysis (Fig. 9) suggested that prior history of delirium was a risk factor for pod in elderly patients with hip fracture (OR = 6.44, 95% CI [2.95–14.06]), due to the large heterogeneity of the indicators, sensitivity analysis was performed and the analysis results (Figure S7, Supplemental Digital Content, https://links.lww.com/MD/R240) suggested that minimal influence on the results.

#### 3.4.10. Publication bias

The current study used funnel plots and the egger test to detect their publication bias, and the funnel plot results (Figures S8–S15, Supplemental Digital Content, https://links.lww.com/MD/R240), where Egger test results for age > 80 (0.001), ASA classification > 4 (0.027), and general anesthesia (0.006) suggest the presence of publication bias, and the rest of the indicators did not have publication bias.

## 4. Discussion

Although numerous meta-analyses have previously explored similar topics, such as Qi YM’s 2022^[17]^ study which included 44 articles, her inclusion criterion was age over 60. In contrast, this study’s inclusion criterion is age over 65, thus constituting a key distinction between our approaches. The study found that age >80 years, ASA score >4, dementia, diabetes mellitus, general anesthesia, hypertension, being male, and a history of delirium were the risk factors for the development of pod in elderly patients undergoing hip surgery. This study was a meta-analysis of the incidence of POD after hip fracture. The overall results showed that the incidence of POD after hip fracture was 24%. Subgroup analysis showed that the incidence of POD was 30% in American patients, while the incidence in Chinese patients was 22% (indicating some differences in the incidence of POD between different countries or regions). Meta-regression analysis was used in this study, which included variables such as year of publication, country, sample size, and mean age of patients. The results of the regression analysis showed that none of the above variables significantly explained the source of heterogeneity, suggesting that there are other under-identified factors affecting the incidence of POD in the current study, such as the level of control of underlying preoperative diseases, intraoperative anesthesia modality, postoperative analgesia management, time point of assessment, and inconsistency in diagnostic criteria.

In the present study, it was found that patients aged > 80 years were more prone to POD, with age, the body’s systems gradually decline, especially the nervous system.^[33]^ Significant degenerative changes in the structure and function of the brain, reduced cerebral blood flow, neuronal loss, and neurotransmitter imbalances lead to reduced cognitive function in the elderly during postoperative recovery.^[34]^ It has been found that older groups of older adults have less cognitive reserve in the brain, are less able to respond to stress in the face of the external environment and surgical interventions and are susceptible to the effects of anesthetic medications and physiological changes. In addition, older adults often suffer from poor sleep quality, underlying diseases, and frailty, all of which provide conditions for pod to occur.^[35]^ To reduce age-induced risks, early cognitive assessment and individualized anesthesia management for elderly patients should be adopted in clinical practice. For example, short-acting anesthetic drugs should be used to reduce the cumulative effects of anesthetic drugs. Postoperatively, measures for cognitive recovery should be implemented as early as possible, such as early physical activity, provision of cognitive training, and regular assessment of the patient’s cognitive function to aid in recovery.^[36]^ The ASA score is used to assess a patient’s general health status, with higher scores indicating poorer underlying health. patients with ASA scores >4 often have multiple underlying medical conditions, such as cardiovascular disease, diabetes mellitus, and hepatic and renal insufficiency, and intraoperative and postoperative metabolic disturbances and organ failure may exacerbate the risk of pod.^[37]^ In addition, these patients often require additional pharmacologic interventions, which may place an additional burden on the brain and increase the chance of delirium. Physiologic abnormalities such as intraoperative hypotension and hypoxemia may also be a contributing factor to delirium. Patients with high ASA scores should be subjected to a more rigorous preoperative evaluation to optimize the management of their underlying disease.^[38]^ Preoperative targeting to improve patients’ cardiopulmonary function and control underlying diseases such as diabetes and blood pressure can significantly reduce postoperative complications. This is consistent with the results of MacLullich et al,^[39]^ in which the incidence of pod was significantly higher in patients with higher ASA scores. Dementia is a group of diseases characterized by a significant decline in cognitive function, commonly including Alzheimer disease and vascular dementia.^[40]^ The nervous system of dementia patients has been severely damaged, and their brains are unable to effectively regulate external stimuli and stress responses. Due to the lack or imbalance of neurotransmitters, dementia patients are less responsive to external medications, environmental changes and surgical procedures, and are prone to POD.^[41]^ Patients with Alzheimer disease, whose lack of neurotransmitters such as acetylcholine in the brain has rendered their cognitive function severely impaired, are more prone to cognitive dysfunction and induced delirium. For patients with dementia, special attention should be paid to their preoperative assessment of cognitive function and postoperative avoidance of drugs that may affect cognitive function, such as benzodiazepines. In addition, a quiet and cozy recovery environment needs to be established after surgery to reduce external interference. Diabetes mellitus has wide-ranging effects on the nervous system and systemic metabolism. Chronic hyperglycemia can cause microangiopathy, especially changes in the blood vessels of the brain, reducing the blood supply to the brain and affecting the function of brain cells. In addition, diabetes can lead to nerve damage and affect the function of peripheral nerves, further exacerbating postoperative cognitive dysfunction. Postoperatively, diabetic patients with large fluctuations in blood glucose, especially hypoglycemia or hyperglycemia, can severely affect the normal function of the nervous system and induce pod.^[42]^ Diabetic patients should strictly control their blood glucose level before surgery and keep it within the normal range to avoid excessive fluctuation of blood glucose after surgery.^[43]^ Postoperatively, blood glucose levels should be closely monitored, and drug regimens should be adjusted in a timely manner to maintain a stable blood glucose status. In addition, the management of diabetes-related complications, such as controlling hypertension and preventing infection, should be strengthened to reduce their impact on postoperative recovery.^[25]^

General anesthesia drugs work through the central nervous system, suppressing brain activity and causing the patient to enter a state of unconsciousness.^[44]^ The effects of anesthetic drugs on the brain may lead to postoperative cognitive dysfunction, especially in the elderly. The slow metabolism of general anesthesia drugs allows anesthesia drugs to accumulate in the body, delaying wakefulness, which may lead to postoperative confusion and cognitive impairment.^[45]^ Physiological changes such as hypotension and hypoxemia that occur during anesthesia may also affect the blood supply to the brain, which in turn may trigger pod. To reduce the effect of anesthesia on pod, appropriate anesthetic regimens should be selected according to the patient’s health status. For elderly patients, short-acting anesthetic drugs should be preferred, and the duration of drug use should be reduced. The patient’s blood pressure and blood oxygen should be kept stable during anesthesia to avoid hypotension and hypoxemia.^[46]^ Hypertension is one of the main causes of cerebrovascular pathology. Chronic high blood pressure can cause small vessel pathology and insufficient blood flow to the brain, which in turn affects cognitive functions of the brain. The autonomic nervous system may be damaged in hypertensive patients, leading to abnormal nerve conduction.^[47]^ Postoperatively, fluctuations in blood pressure and the use of anesthetic drugs may further exacerbate the lack of blood flow to the brain and increase the risk of pod. Hypertensive patients should have their blood pressure strictly controlled and kept within the normal range before surgery. During anesthesia, the patient’s blood pressure should be monitored to avoid fluctuations of hypotension or hypertension. Postoperatively, blood pressure should be measured regularly, and appropriate medication adjustments should be taken to ensure blood pressure stabilization.^[48]^ It has been shown that male patients experiencing surgical interventions are at a higher risk for pod than females due to physiologic, psychological, and biological differences. Men tend to experience a more pronounced stress response postoperatively, which may be related to higher metabolic rates, hormone levels, and other factors in men,^[49]^ which further supports the findings of the current study. Patients with a previous history of delirium have already experienced cognitive impairment of the neurological system of the brain, with poorer neurotransmitter homeostasis and cerebral blood flow stability. As a result, these patients are at higher risk of developing delirium postoperatively.^[50]^ A history of delirium may lead to long-term impairment of brain function and increase the likelihood of postoperative cognitive dysfunction. Patients with a history of delirium should be monitored more rigorously preoperatively and postoperatively. A combination of cognitive function assessment, environmental interventions, and medication management is used to minimize recurrence of delirium.^[51]^

To address the high-risk factors for POD in elderly patients after hip arthroplasty, nursing staff should develop a personalized care plan. Preoperative systematic health assessment should be carried out for patients, including cognitive function, nutritional status and control of underlying diseases, focusing on high-risk groups such as advanced age, comorbid dementia, diabetes mellitus, and hypertension. During the operation, the anesthesiologist should be cooperated with the patient to continuously monitor the patient’s blood pressure and blood oxygen saturation, to detect and correct hypotension or hypoxemia in time, and to reduce the impact on the cerebral blood flow. After surgery, nursing staff should strengthen the patient’s environmental management, provide a quiet, comfortable, and appropriately lighted recovery environment, reduce noise stimulation and frequent interference, and promote the patient’s cognitive recovery. Meanwhile, nursing staff should conduct regular cognitive function screening to recognize early manifestations of POD early and take non-pharmacological interventions, such as early out-of-bed activities, cognitive training, and sleep quality promotion. In addition, for patients with a history of previous delirium, closer observation and nursing interventions should be implemented to reduce the risk of recurrent POD.

## 5. Future clinical guidance

Future research directions should further explore the impact of systematic nursing interventions on the incidence of POD, especially the precise nursing strategies developed for different risk factors. At the same time, a multidisciplinary teamwork model can be explored, such as collaboration among nursing, anesthesia, internal medicine, and rehabilitation, to establish a comprehensive patient-centered management pathway. In addition, training of nursing staff on POD risk identification and intervention skills should be strengthened to improve the nursing team’s ability in early detection and management of POD. With the development of information technology, in the future, we can also try to apply emerging technologies, such as intelligent monitoring systems and digital cognitive training platforms, to assist in the recovery of cognitive function and the prevention of POD in elderly patients after surgery.

## 6. Limitations

There are some limitations in this meta-analysis. First, most of the included studies were retrospective, with some selection bias, and there was a high degree of heterogeneity in design, sample size, and patient population across studies, which may have affected the accuracy and generalizability of the results. Second, since the data of most of the studies were observational, there might be uncontrolled confounding factors and potential bias could not be completely excluded. Third, the quality of some of the included studies was not high, and despite our bias assessment, there may be systematic bias that affects the results. In addition, although a random-effects model was used to deal with heterogeneity, different assessment criteria, follow-up times, and measurement tools may have led to some outcome bias. Finally, regarding the analysis of certain risk factors, the limited number of studies may not have sufficiently revealed their potential impact, and factors with greater heterogeneity may have had some impact on the robustness of the results. Therefore, although this study provides valuable conclusions, more high-quality prospective randomized controlled trials are needed to validate these findings and further explore their clinical applications.

## 7. Conclusion

In this study, we assessed the relationship between multiple factors and pod in elderly hip arthroplasty by meta-analysis, which showed that age >80 years, ASA score >4, dementia, diabetes mellitus, general anesthesia, hypertension, being male, and a history of delirium were all risk factors for pod. Despite some heterogeneity, the results of the sensitivity analysis were relatively stable, suggesting that these factors are clinically important in elderly patients. Therefore, in clinical practice, individualized preoperative assessment and postoperative management should be performed for these high-risk patients to reduce the risk of pod and improve patient prognosis. However, considering the heterogeneity of studies and the quality of some studies, more high-quality prospective studies are needed in the future to further validate these results and to explore more precise interventions.

## Author contributions

**Conceptualization:** Yue Hu, Wen Tang.

**Data curation:** Yue Hu, Wen Tang, Xiuzhen Jiang.

**Formal analysis:** Xiuzhen Jiang.

**Funding acquisition:** Yue Hu, Wen Tang, Xiuzhen Jiang.

**Investigation:** Yue Hu, Wen Tang.

**Methodology:** Yue Hu, Xiuzhen Jiang.

**Project administration:** Xiuzhen Jiang.

**Resources:** Wen Tang, Xiuzhen Jiang.

**Software:** Yue Hu, Wen Tang, Xiuzhen Jiang.

**Supervision:** Yue Hu.

**Validation:** Yue Hu, Xiuzhen Jiang.

**Visualization:** Yue Hu, Wen Tang.

**Writing – original draft:** Yue Hu, Wen Tang, Xiuzhen Jiang.

**Writing – review & editing:** Xiuzhen Jiang.

## Supplementary Material


