# Supplementary material for: Prevalence and risk factors for postoperative delirium after hip fracture in the elderly: A systematic review and meta-analysis
Source: Medicine (Baltimore). 2026 Jan 23;105(4):e47296. doi: 10.1097/MD.0000000000047296 (PMC12851674; doi:10.1097/MD.0000000000047296)
Supplement: Supplementary file 1 [file medi-105-e47296-s001.pdf]

Figure S1 The prevalence of the meta-analysis

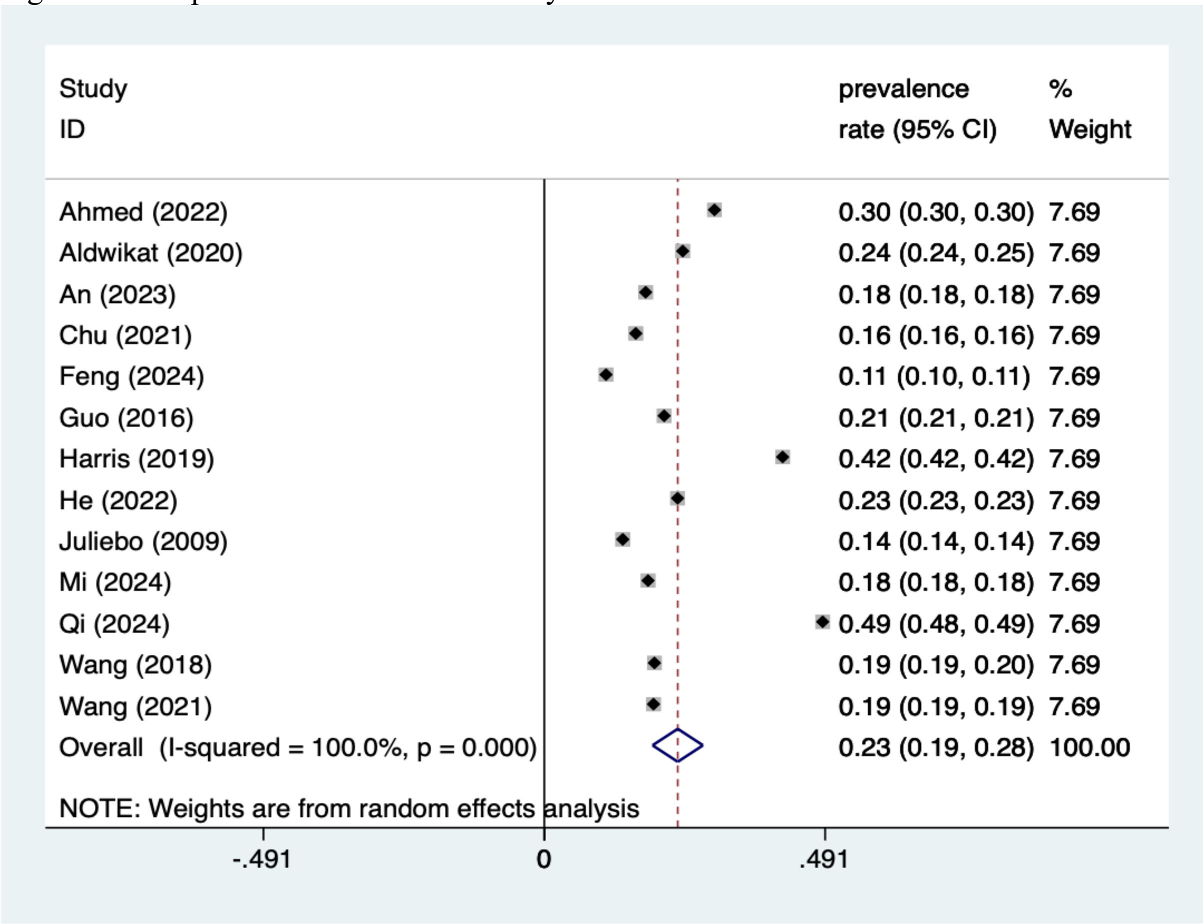

Figure s2 Meta-analysis of the prevalence rate in countries

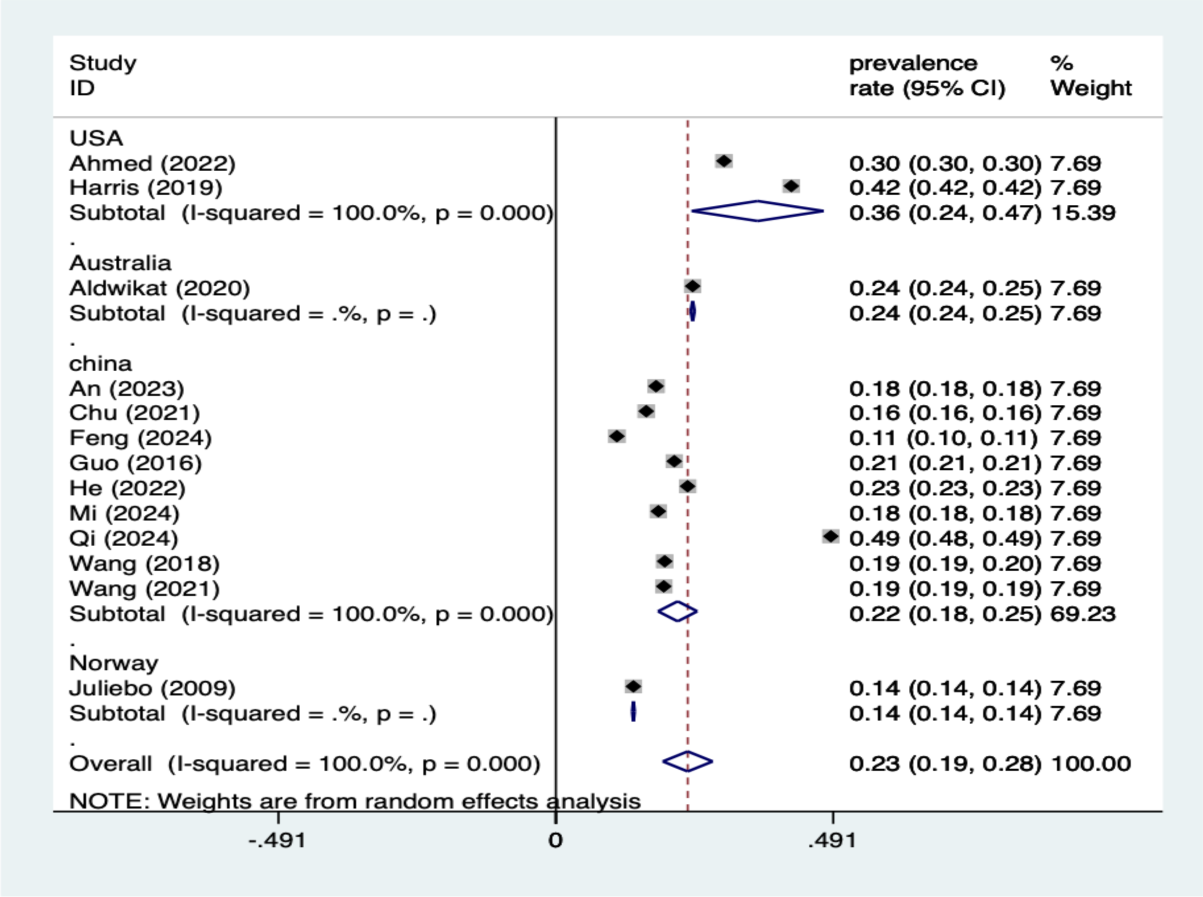

Figure S3 Sensitivity analysis of Age>80

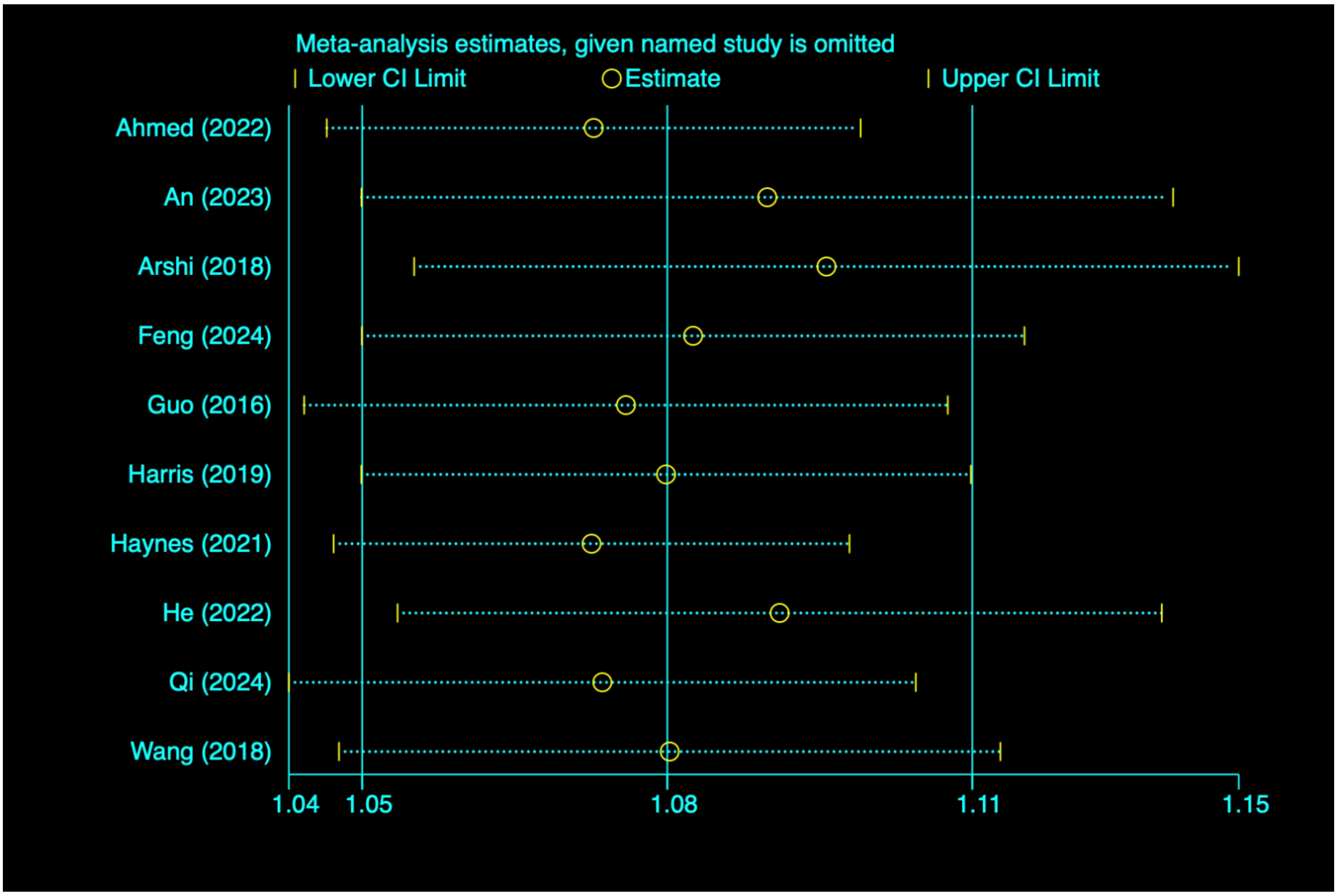

Figure S4 Sensitivity analysis of ASA classification>4

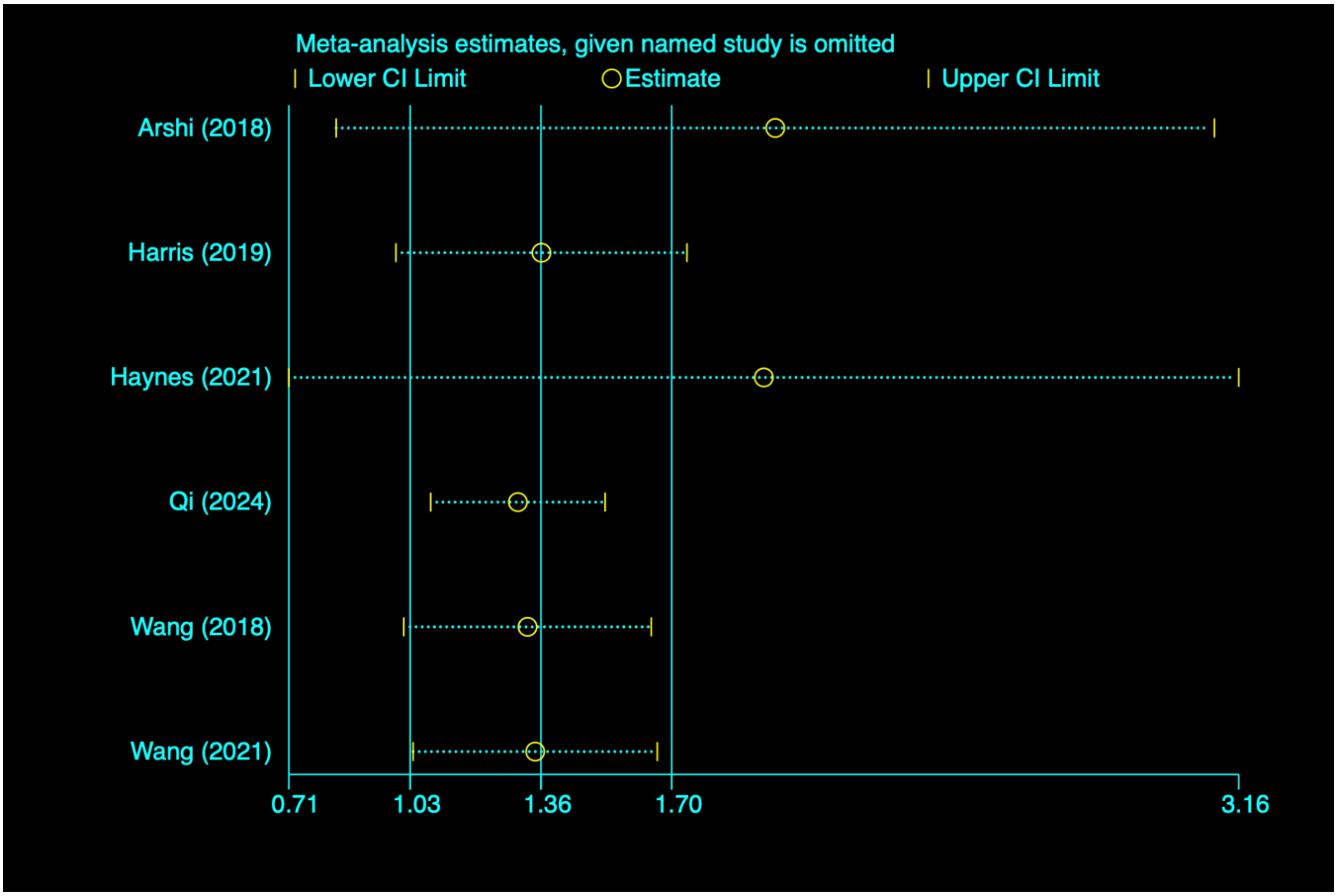

Figure S5 Sensitivity analysis of diabetes

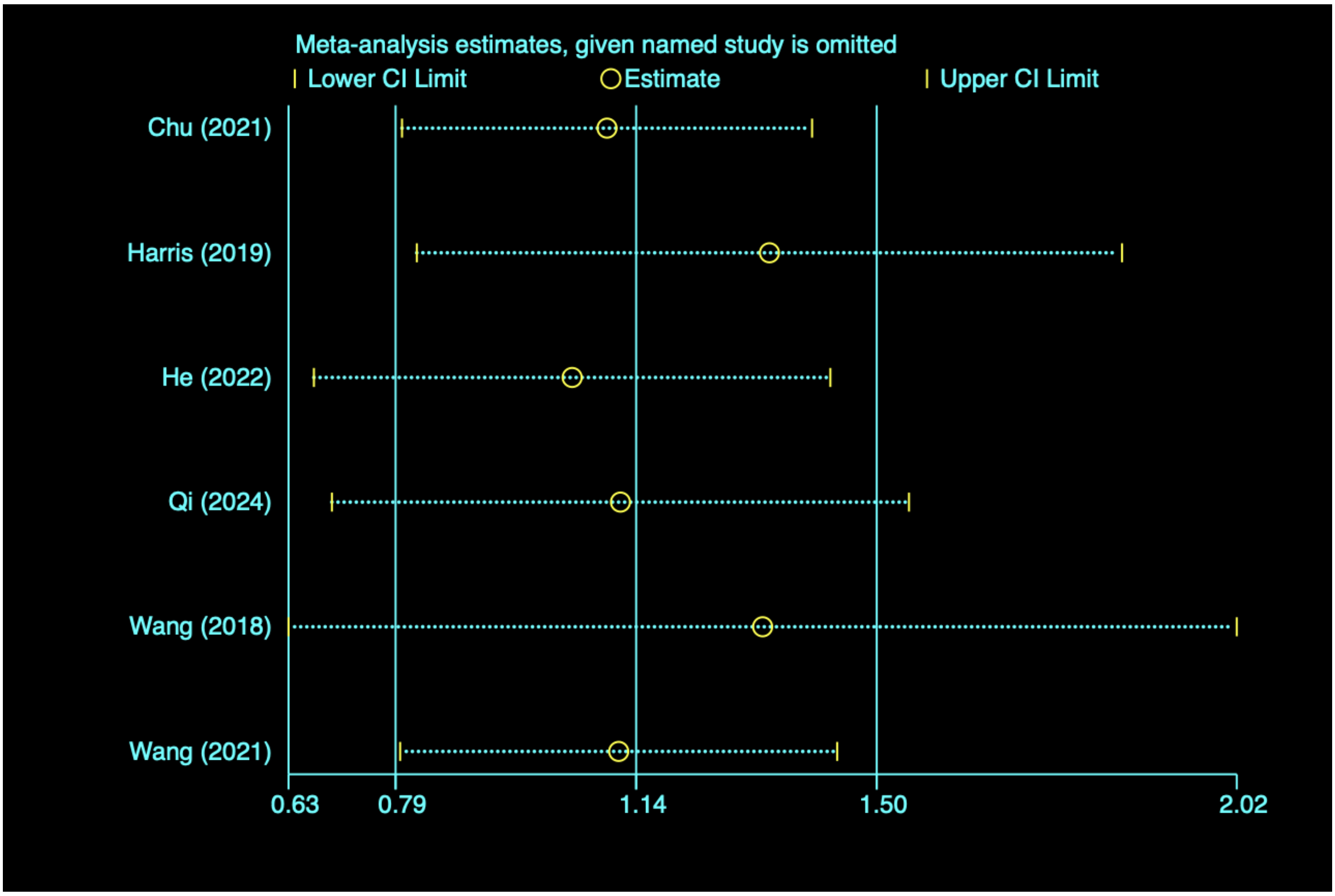

Figure S6 Sensitivity analysis of general anesthesia

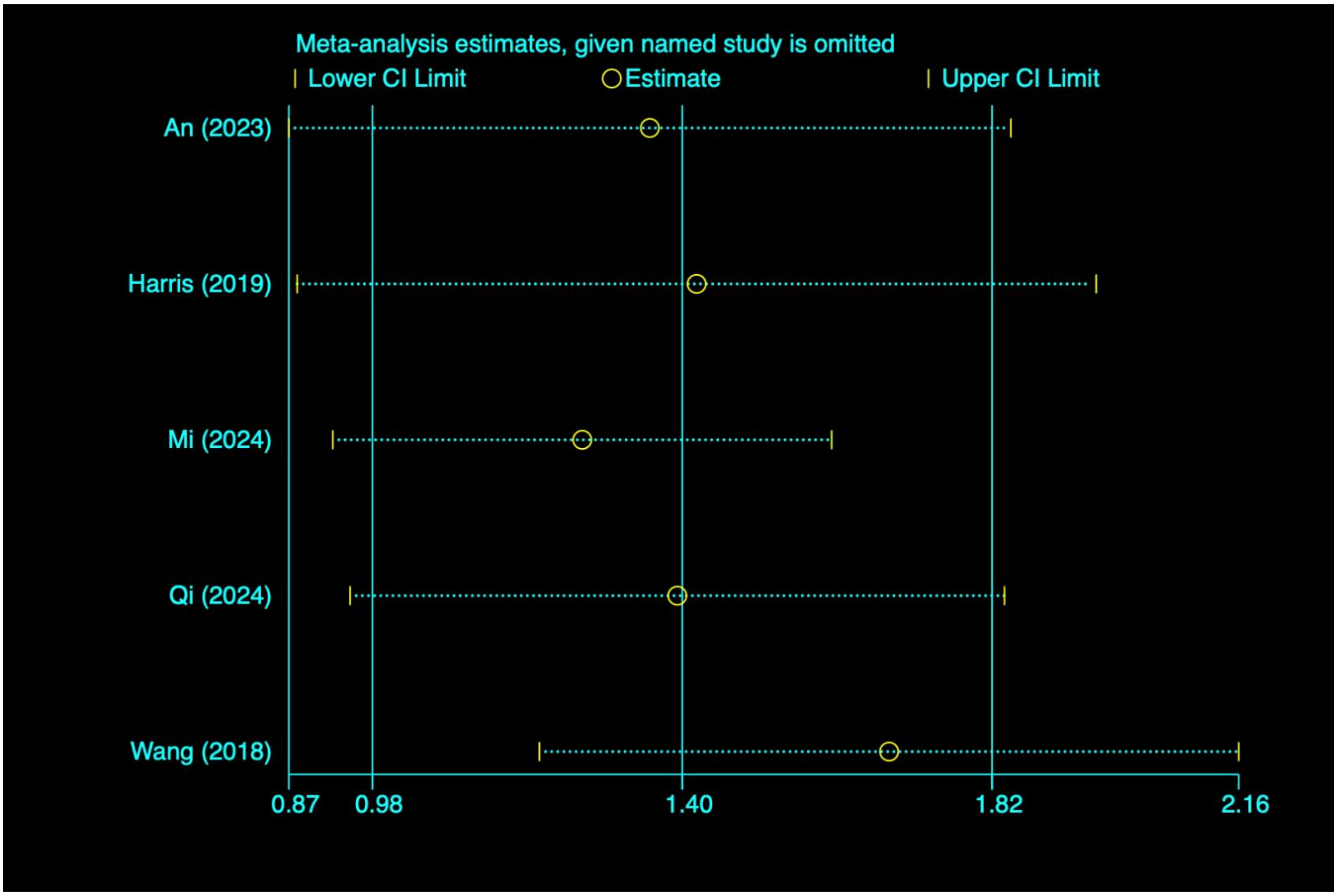

Figure S7 Sensitivity analysis of prior history of delirium

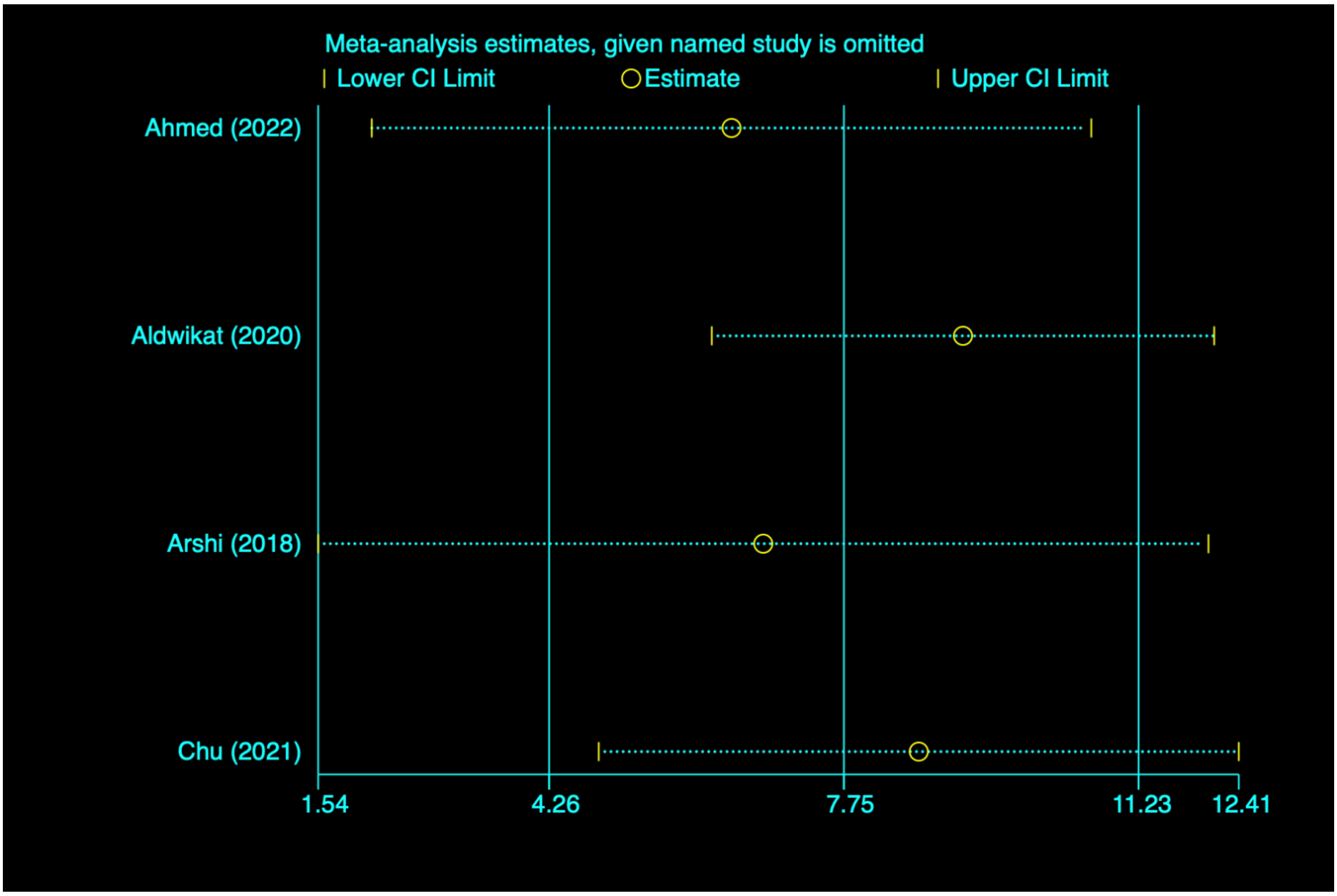

Figure S8 Age>80 meta-analysis funnel plot

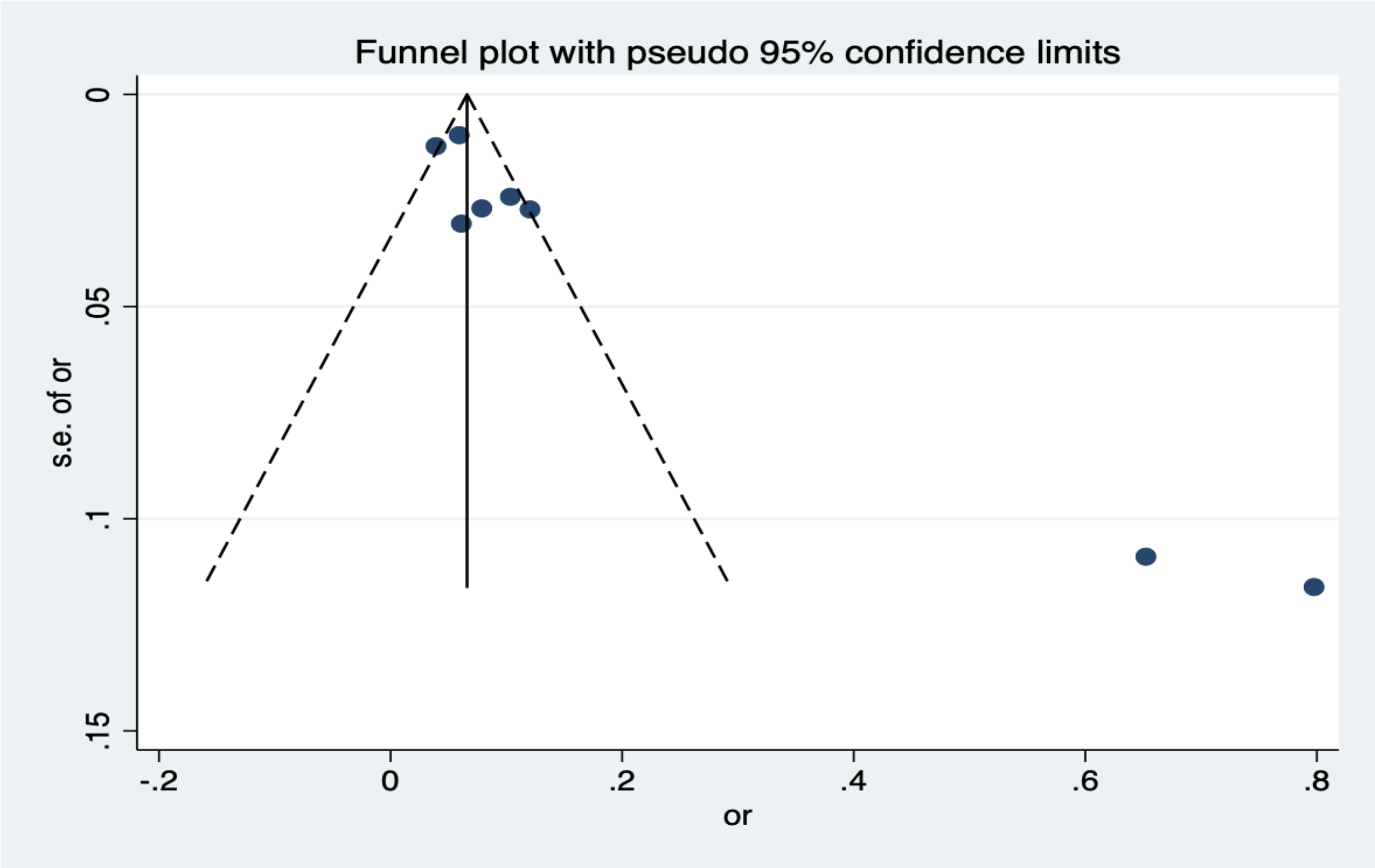

Figure S9 ASA classification>4 meta-analysis funnel plot

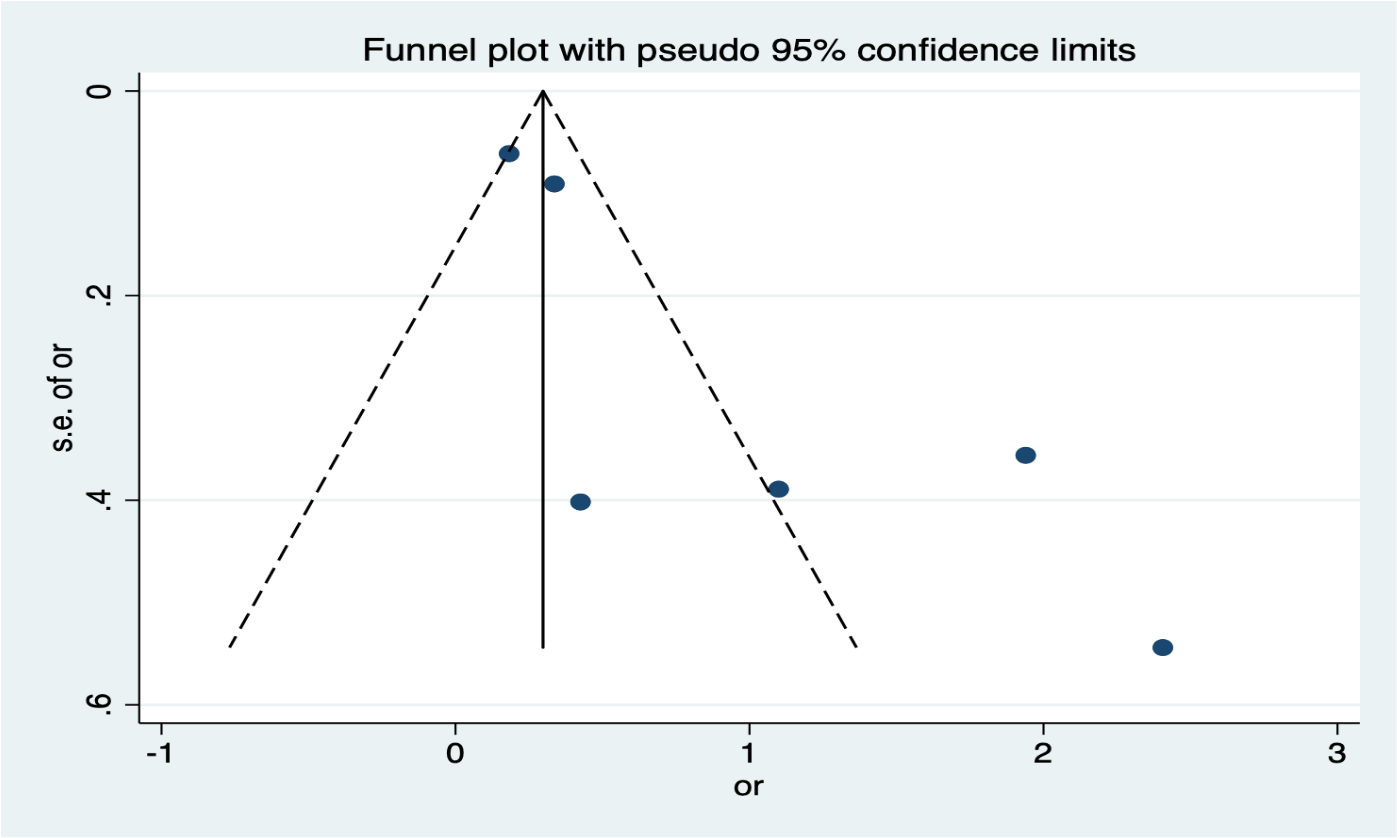

Figure S10 dementia meta-analysis funnel plot

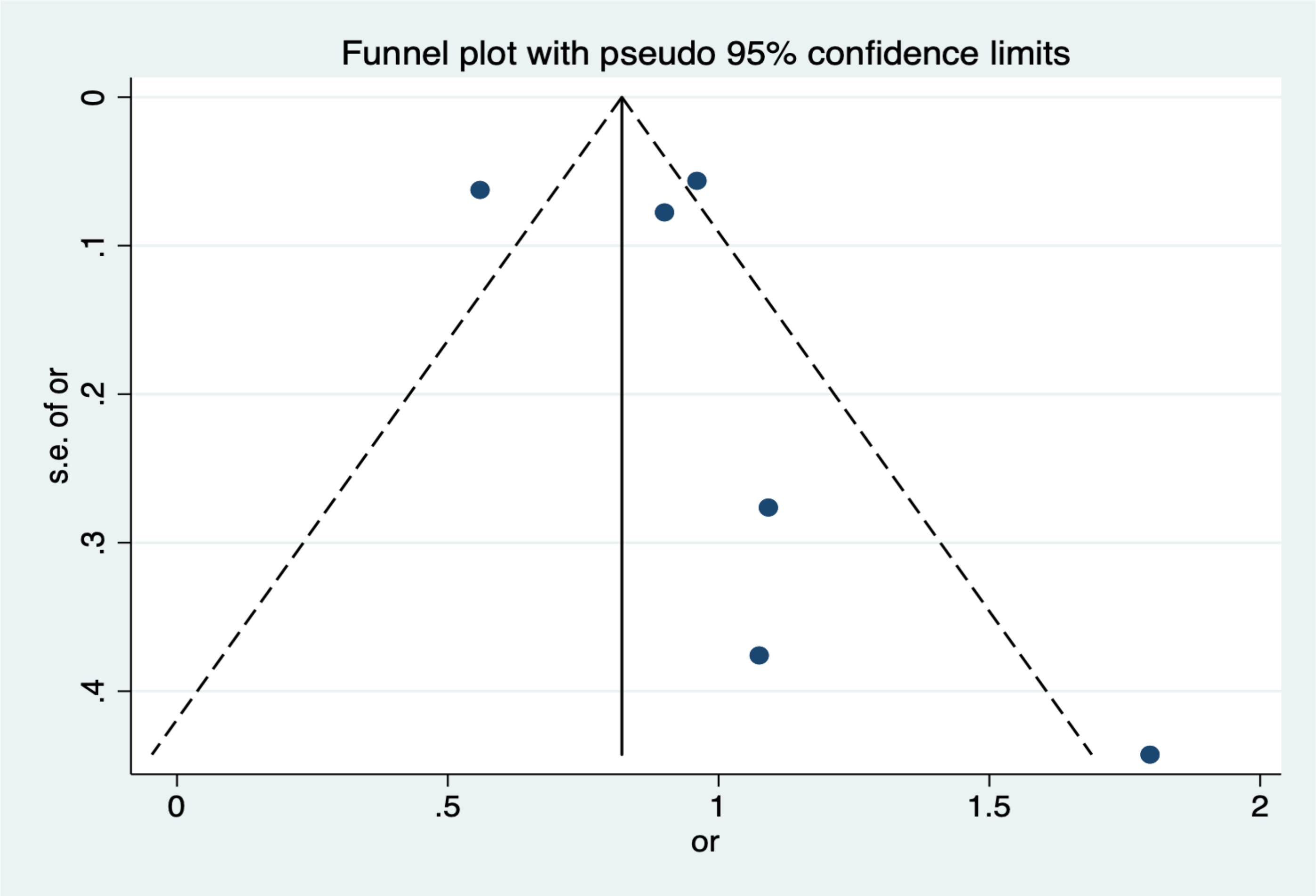

Figure S11 diabetes meta-analysis funnel plot

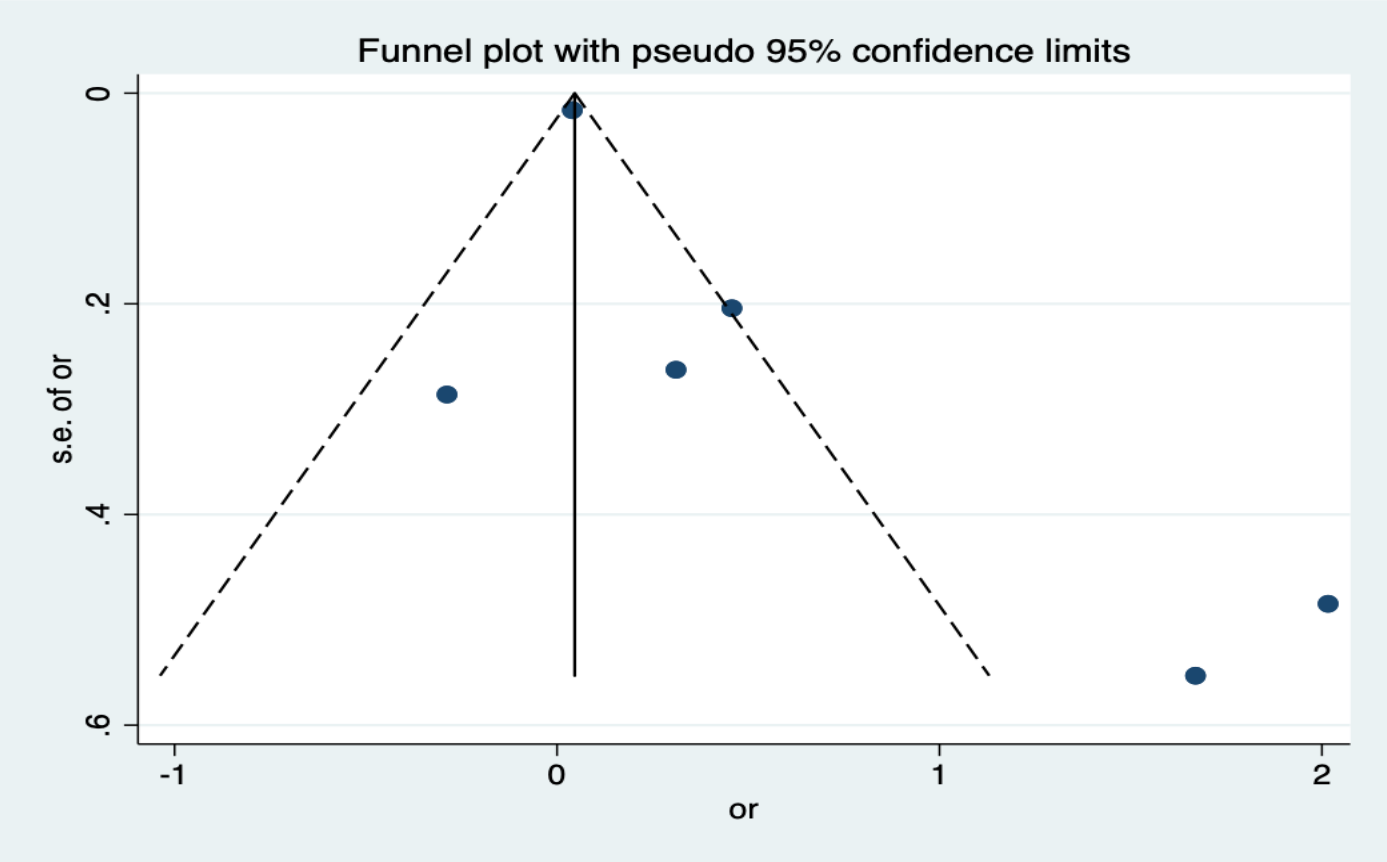

Figure S12 general anesthesia meta-analysis funnel plot

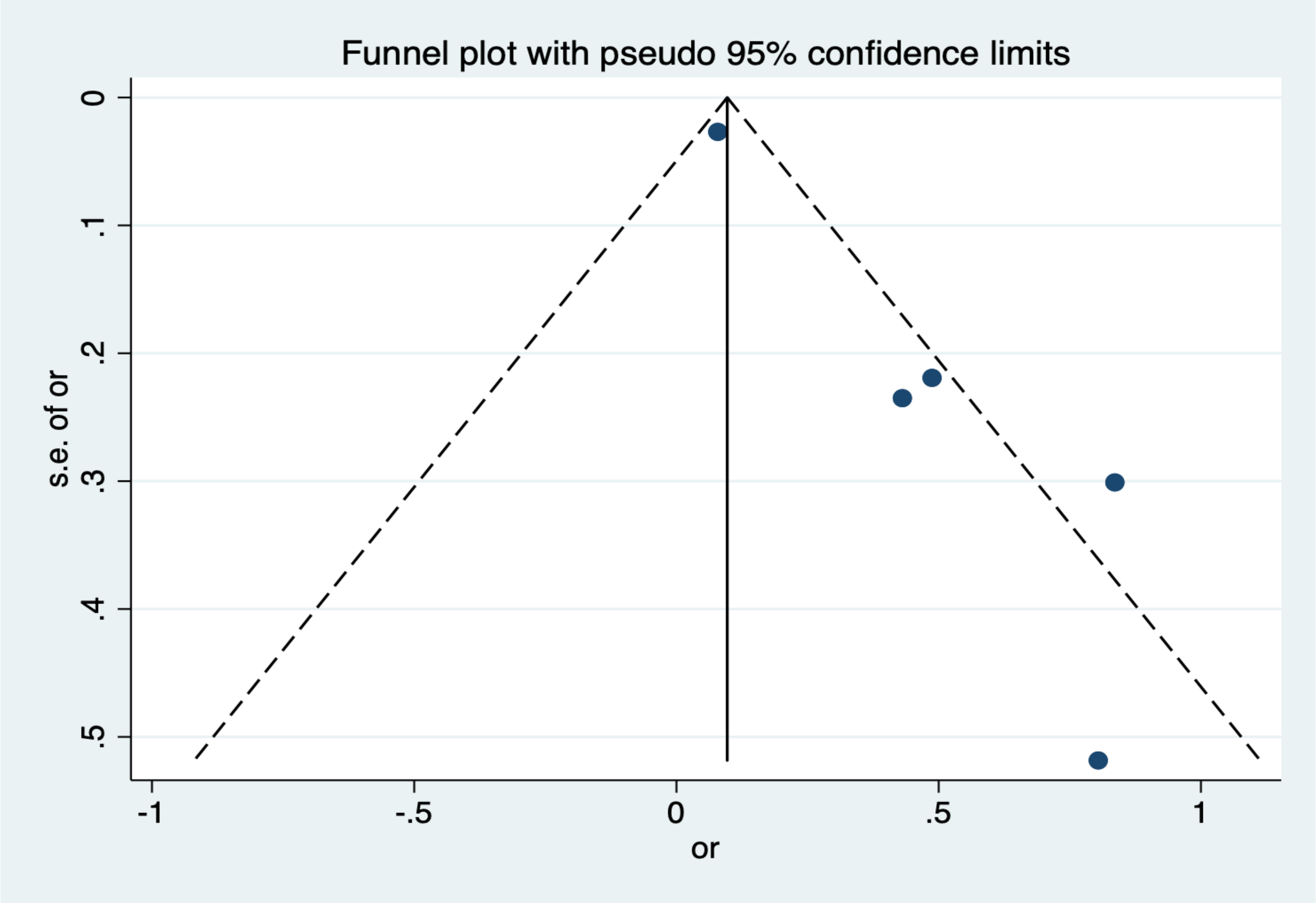

Figure S13 hypertension meta-analysis funnel plot

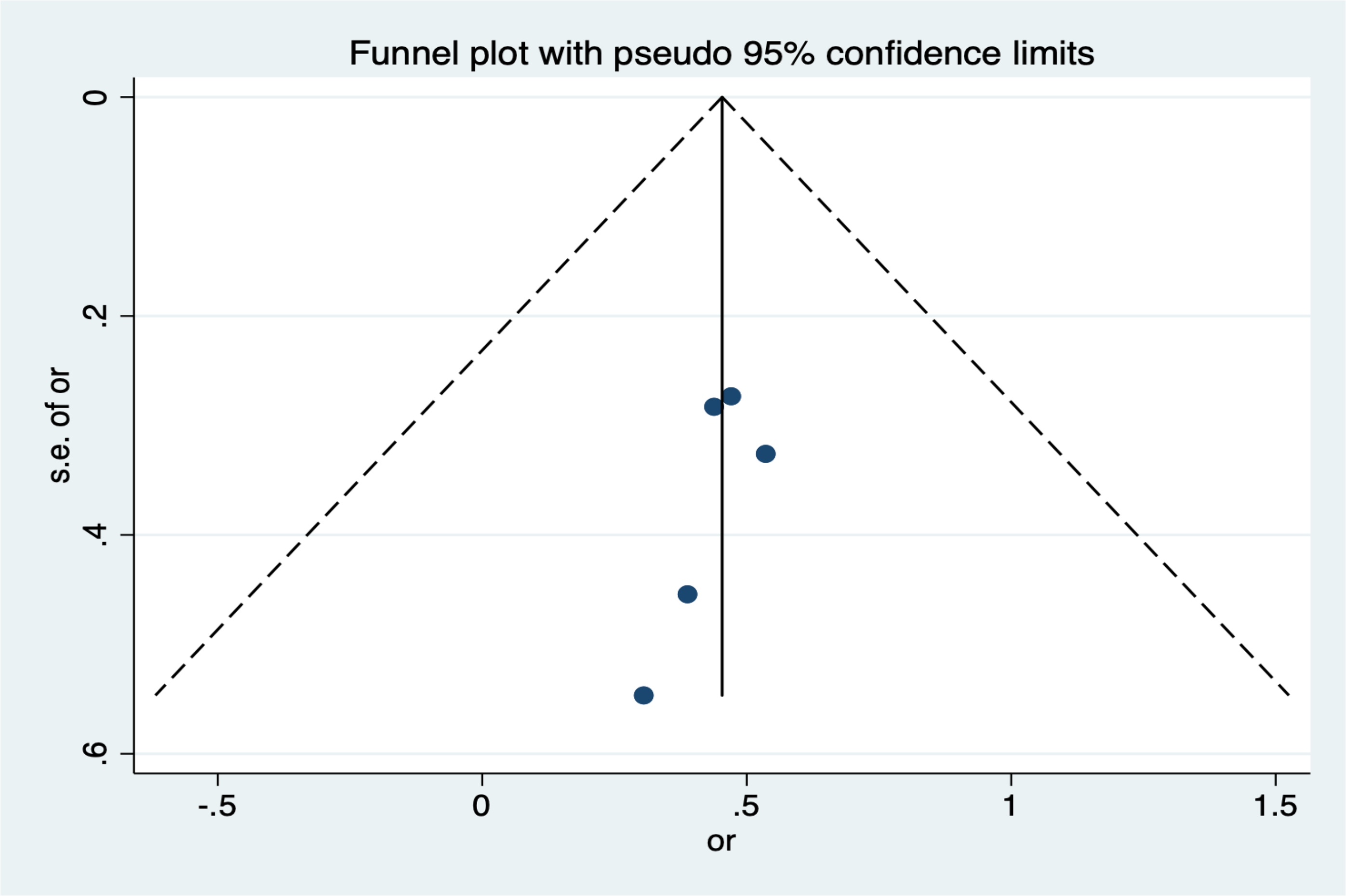

Figure S14 male meta-analysis funnel plot

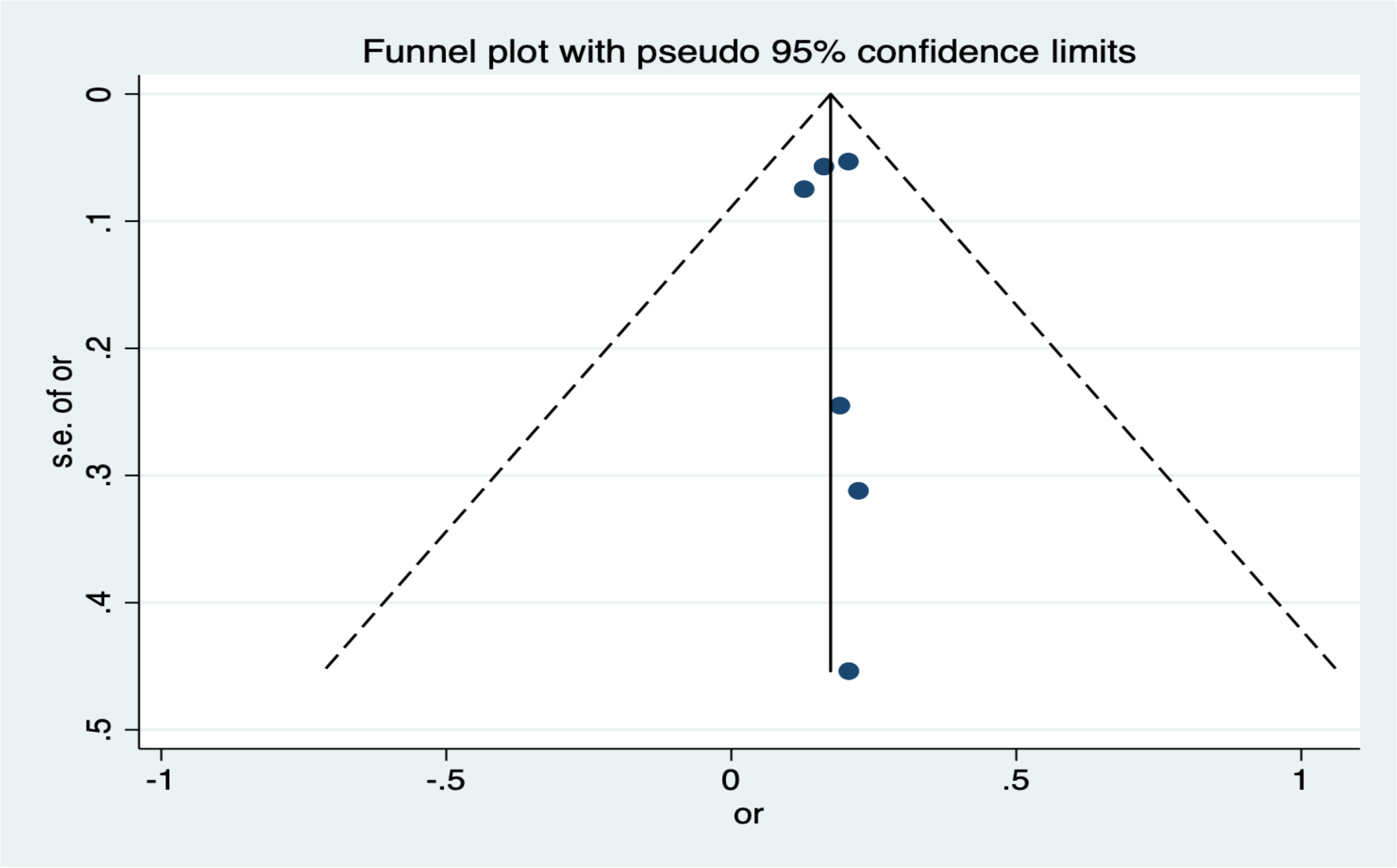

Figure S15 prior history of delirium meta-analysis funnel plot

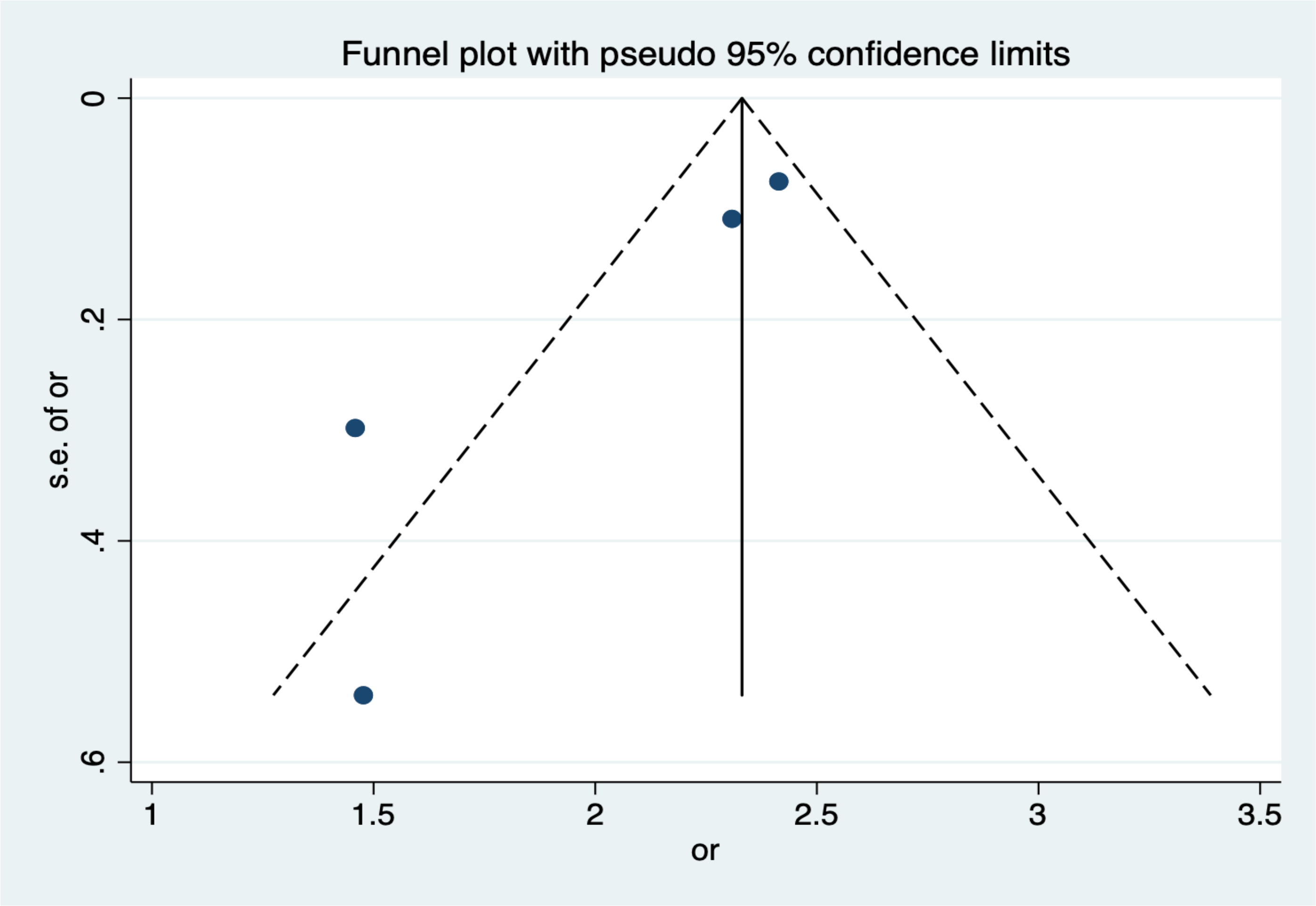

Table S1 specific search strategy

(((("Aged"[Mesh]) OR ((Aged[Title/Abstract]) OR (Elderly[Title/Abstract]))) AND (("Hip Fractures"[Mesh]) OR ((((((((((((((((((Hip Fractures[Title/Abstract]) OR (Fractures, Hip[Title/Abstract])) OR (Intertrochanteric Fractures[Title/Abstract])) OR (Fractures, Intertrochanteric[Title/Abstract])) OR (Subtrochanteric Fractures[Title/Abstract])) OR (Fractures, Subtrochanteric[Title/Abstract])) OR (Trochanteric Fractures[Title/Abstract])) OR (Fractures, Trochanteric[Title/Abstract])) OR (Trochlear Fractures, Femur[Title/Abstract])) OR (Femur Trochlear Fracture[Title/Abstract])) OR (Femur Trochlear Fractures[Title/Abstract])) OR (Fracture, Femur Trochlear[Title/Abstract])) OR (Fractures, Femur Trochlear[Title/Abstract])) OR (Trochlear Fracture, Femur[Title/Abstract])) OR (Femoral Trochlear Fractures[Title/Abstract])) OR (Femoral Trochlear Fracture[Title/Abstract])) OR (Fracture, Femoral Trochlear[Title/Abstract])) OR (Fractures, Femoral Trochlear[Title/Abstract])) OR (Trochlear Fracture, Femoral[Title/Abstract])) OR (Trochlear Fractures, Femoral[Title/Abstract])))) AND (("Emergence Delirium"[Mesh]) OR ((((((Emergence Delirium[Title/Abstract]) OR (Postoperative Delirium[Title/Abstract])) OR (Delirium, Postoperative[Title/Abstract])) OR (Post-Operative Delirium[Title/Abstract])) OR (Delirium, Post-Operative[Title/Abstract])) OR (Post Operative Delirium[Title/Abstract])))) AND (("Risk Factors"[Mesh]) OR ((((((((((((((((((Risk Factors[Title/Abstract]) OR (Factor, Risk[Title/Abstract])) OR (Risk Factor[Title/Abstract])) OR (Population at Risk[Title/Abstract])) OR (Populations at Risk[Title/Abstract])) OR (Risk Scores[Title/Abstract])) OR (Risk Score[Title/Abstract])) OR (Score, Risk[Title/Abstract])) OR (Risk Factor Scores[Title/Abstract])) OR (Risk Factor Score[Title/Abstract])) OR (Score, Risk Factor[Title/Abstract])) OR (Health Correlates[Title/Abstract])) OR (Correlates, Health[Title/Abstract])) OR (Social Risk Factors[Title/Abstract])) OR (Factor, Social Risk[Title/Abstract])) OR (Factors, Social Risk[Title/Abstract])) OR (Risk Factor, Social[Title/Abstract])) OR (Risk Factors, Social[Title/Abstract])) OR (Social Risk Factor[Title/Abstract])))

Table S2 Meta-regression analysis

	TYPE	Coef	Std. Err	P	95%CI
Prevalence	Year	0.005	0.007	0.525	（-.011， 0.020）
	Country	0.105	0.147	0.490	(-0.219,0.428)
	sample size	0.135	0.499	0.791	(-0.944, 0.001)
	mean age	0.007	0.006	0.231	(-0.005, 0.020)
